# The blackgrass genome reveals patterns of non‐parallel evolution of polygenic herbicide resistance

**DOI:** 10.1111/nph.18655

**Published:** 2023-01-12

**Authors:** Lichun Cai, David Comont, Dana MacGregor, Claudia Lowe, Roland Beffa, Paul Neve, Christopher Saski

**Affiliations:** ^1^ Department of Plant and Environmental Sciences Clemson University Clemson SC 29634 USA; ^2^ Protecting Crops and the Environment Rothamsted Research Harpenden, Hertfordshire AL5 2JQ UK; ^3^ Bayer Crop Sciences Industriepark Höchst 65926 Frankfurt am Main Germany; ^4^ Königsteiner Weg 4 65835 Liederbach Germany; ^5^ Department of Plant and Environmental Sciences University of Copenhagen Højbakkegård Allé 13 Tåstrup 2630 Denmark

**Keywords:** blackgrass (*Alopecurus myosuroides*), herbicide resistance, parallel evolution, polygenic trait, quantitative genetics, rapid plant adaptation, weed evolution, weed genomics

## Abstract

- Globally, weedy plants are a major constraint to sustainable crop production. Much of the success of weeds rests with their ability to rapidly adapt in the face of human‐mediated management of agroecosystems. *Alopecurus myosuroides* (blackgrass) is a widespread and impactful weed affecting agriculture in Europe.
- Here we report a chromosome‐scale genome assembly of blackgrass and use this reference genome to explore the genomic/genetic basis of non‐target site herbicide resistance (NTSR). Based on our analysis of F2 seed families derived from two distinct blackgrass populations with the same NTSR phenotype, we demonstrate that the trait is polygenic and evolves from standing genetic variation.
- We present evidence that selection for NTSR has signatures of both parallel and non‐parallel evolution. There are parallel and non‐parallel changes at the transcriptional level of several stress‐ and defence‐responsive gene families. At the genomic level, however, the genetic loci underpinning NTSR are different (non‐parallel) between seed families.
- We speculate that variation in the number, regulation and function of stress‐ and defence‐related gene families enable weedy species to rapidly evolve NTSR via exaptation of genes within large multi‐functional gene families. These results provide novel insights into the potential for, and nature of plant adaptation in rapidly changing environments.

## Introduction

Human‐mediated environmental change is driving rapid evolutionary responses in the global biota (Palumbi, 2001; Hendry *et al*., 2011) and it is important to understand the outcome of these changes in natural and agricultural plant populations and communities. Reference genomes offer glimpses into the adaptive potential of plants when challenged with novel stresses, while agricultural weeds have been proposed as ideal models to address fundamental questions in plant ecology and evolution (Neve *et al*., 2009; Vigueira *et al*., 2013; Kreiner *et al*., 2018; Baucom, 2019; Mahaut *et al*., 2020).

Herbicide use has become a mainstay of weed management. Unsurprisingly, heavy reliance on herbicides has resulted in the rapid and widespread evolution of resistance, making herbicide resistance a widely studied weedy trait (Heap, 2014; Gould *et al*., 2018). Two ‘types' of herbicide resistance are recognized (Powles & Yu, 2010; Gaines *et al*., 2020). Target site resistance (TSR) refers to the modification of the sequence, copy number and/or expression of the gene encoding the herbicide target enzyme. Non‐target site resistance (NTSR) encompasses a range of mechanisms that limit herbicide delivery to its site of action. Typically, NTSR is inherited in a quantitative manner, but despite some advances in identifying and/or validating causal loci (Cummins *et al*., 2013; Delye, 2013; Tetard‐Jones *et al*., 2018; Franco‐Ortega *et al*., 2021; Han *et al*., 2021), efforts to discern the genomic basis and evolutionary dynamics of this trait have been hampered by lack of access to genomic resources.

The widespread evolution of herbicide resistance is an emblematic example of repeated (or convergent) evolution of plant defence in the face of an extreme, novel selection pressure (Baucom, 2016). In general, TSR has provided an example of genetic parallelism (Martin & Orgogozo, 2013) where the convergent evolution of resistance is underpinned by parallel patterns of selection at single major loci (Powles & Yu, 2010; Gaines *et al*., 2020). The genetic basis of NTSR is not fully resolved, but current evidence suggests that this trait is polygenic, that the genomic architecture of NTSR may be determined by selection at parallel and non‐parallel genetic loci (Van Etten *et al*., 2020; Kreiner *et al*., 2021), via co‐option (or exaptation) of standing genetic variation in plant stress‐ and defensive‐responsive pathways (Hawkins *et al*., 2019). Addressing these questions through studies of the genomic basis of NTSR has power to answer fundamental questions about the importance of genetic parallelism and non‐parallelism, genomic constraint, genetic background (contingency) and standing genetic variation in rapid plant adaptation to a novel environmental stress (Allen Orr, 2005a,b; Losos, 2011; Lobkovsky & Koonin, 2012; Bolnick *et al*., 2018).

The diploid, allogamous grass, *Alopecurus myosuroides* (blackgrass) is native to the Eastern Mediterranean and West Asia (Bulcke, 1975) but is now a widespread and impactful weed in agricultural crops in much of Europe (Menchari *et al*., 2007; Rosenhauer *et al*., 2013; Hicks *et al*., 2021) and in China (Liu *et al*., 2021). Blackgrass populations are prone to the rapid and widespread evolution of herbicide resistance. In a nationwide survey in England, most blackgrass populations exhibited resistance to multiple herbicide modes of action (Hicks *et al*., 2018). Resistance was conferred by coexisting TSR and NTSR mechanisms, with evidence that historical herbicide‐use regimes favoured the evolution of the NTSR (Comont *et al*., 2020). Herbicide‐resistant blackgrass is estimated to cost UK farmers £0.4 billion per year (Varah *et al*., 2020) and there is no evidence for fitness costs for any of a variety of life‐history traits associated with NTSR (Comont *et al*., 2022).

Access to genomes and genomic resources for weed species will greatly enhance the capacity to unravel contemporary adaptation in economically and ecologically important weedy plant species (Ravet *et al*., 2018). Here, we present a high‐quality reference genome for blackgrass. We use these genome resources to reveal that patterns of convergent evolution of organismal‐ (whole plant assays) and gene expression‐based phenotypes for NTSR‐based resistance are conferred by non‐parallel changes at multiple genetic loci distributed widely throughout the blackgrass genome.

## Materials and Methods

### Plant materials for genome sequencing and annotation

*Alopecurus myosuroides* L. (blackgrass) seeds collected in 2017 from section 8 of the Rothamsted ‘Broadbalk’ long‐term experiment (Moss *et al*., 2004) were used to select an individual blackgrass plant for genome sequencing. Established in 1843, these field plots have never received herbicide application, and extensive testing of this population (Rothamsted, Hertfordshire, UK) over the last 20 yr has confirmed that it remains susceptible to all herbicides, representing a true wild‐type blackgrass genotype. In addition, two field‐collected blackgrass seed populations (Peldon and Lola91) previously characterized as being strongly NTSR to acetyl‐CoA carboxylase (ACCase) inhibiting herbicides were used to generate F_2_ seed families (named CC2 and CC5, respectively) for quantitative trait loci (QTL)‐seq and RNA‐seq analyses. Detailed protocols for the selection of a single herbicide‐sensitive plant for genome sequencing and for the development of CC2 and CC5 seed families are presented in Notes S1.

### Genome survey

A previous study has reported that blackgrass has seven chromosomes (Johnsson, 1944). In our study, genome size was estimated through flow cytometry and *k*‐mer‐based analysis. Flow cytometry was conducted on four field‐collected blackgrass populations (the Rothamsted, Lola91, and Peldon populations used within this study, plus a further herbicide‐susceptible population). Genome size estimates were generated for three replicate plants from each of these populations, against a known standard of the plant *Allium schoenoprasum*. Using these data, the blackgrass genome size was estimated as 3312–3423 Mb. *K*‐mer‐based analysis from Illumina sequencing data of the Rothamsted population indicated a genome size of 3400–3550 Mb. We also estimated the heterozygosity and repeat content of the blackgrass genome with gce package (https://github.com/BioInfoTools/GCE), the results suggest the blackgrass genome exhibits high levels of heterozygosity (1.52%) and repeat content (84.2%).

### Genome sequencing

A mix of single‐molecule and short read sequencing data was collected for *de novo* genome assembly. These data include 513 Gb (144× coverage) PacBio continuous long reads, 860 Gb (241× coverage) BioNano optical maps, 126 Gb (35× coverage) Hi‐C reads and 291 Gb (81× coverage) Illumia short reads. Detailed protocols for sequencing and assembly of the blackgrass genome are presented in Notes S1.

### Genome assembly

A *de novo* assembly of PacBio long reads into contigs was performed with Mecat2 (Xiao *et al*., 2017). This produced 12 107 contigs with an N50 of 0.9 Mb and a total size of 4906 Mb. The assembled contigs were polished with PacBio long reads via Arrow (https://github.com/PacificBiosciences/SMRT‐Link) and Illumina short reads with Pilon (v.1.20) (Walker *et al*., 2014). Polished contigs were repeat marked using WindowMasker (Morgulis *et al*., 2006) and then haplotype merged using HaploMerger2 (Huang *et al*., 2017) to address the high heterozygosity of the blackgrass genome. BioNano data were first filtered for molecule length (> 150 kb) and then aligned to primary contigs to select mapped molecules for *de novo* assembly to obtain the BioNano optical maps. The primary contigs and BioNano maps were combined to produce the base hybrid scaffold assembly. The Hi‐C reads were aligned to the base assembly using the Juicer pipeline (Durand *et al*., 2016a). Hybrid scaffolds were then further scaffolded using the 3D‐DNA pipeline (Dudchenko *et al*., 2017). The results were manually examined using the Juicebox Assembly Tools, an assembly‐specific module in the Juicebox visualization system (Durand *et al*., 2016b). The Hi‐C scaffolding resulted in seven pseudomolecule chromosomes. Assembly gaps were identified and filled with Cobbler (v.0.6.1) (Warren, 2016). The final assembly was polished again with PacBio long reads via Arrow and Illumina short reads via Pilon (Walker *et al*., 2014). Detailed methods are presented in Notes S1.

### Genome assembly quality assessment

The quality of the genome assembly was evaluated by the following analyses: (1) The Illumina short reads used for polishing were mapped to the genome assembly using Bwa‐Mem, and the mapping rate and genome coverage were examined. (2) The assembly was assessed for single‐copy gene ortholog content with Busco (v.4.0.1) (Simao *et al*., 2015) using the embryophyta_odb10 database. (3) The long terminal repeat (LTR) assembly Index (Ou *et al*., 2018) was calculated. (4) Correlation of the assembled chromosome length to the cytogenic chromosome length (Johnsson, 1944) was examined.

### Genome annotation

A comprehensive non‐redundant repeat library for the blackgrass genome was built using EDTA, a *de novo* transposable element (TE) annotator that integrates structure‐ and homology‐based approaches for TE identification (Ou *et al*., 2019). The EDTA pipeline incorporates LTRharvest, the parallel version of Ltr_Finder, Ltr_retriever, Grf, Tir‐Learner, HelitronScanner and RepeatModeler as well as customized filtering scripts. Genome‐wide prediction of ncRNAs, such as rRNA, small nuclear RNA and miRNA, was performed using the Infernal software (Nawrocki *et al*., 2009) to the Rfam database. The tRNA genes were predicted using tRNAscan‐SE (Lowe & Eddy, 1997).

Protein‐coding genes were predicted by a combination of *de novo* prediction, homology‐based and transcriptome‐based strategies. Snap (Korf, 2004), Augustus (Stanke *et al*., 2006) and GeneMark (Lomsadze *et al*., 2005) were used for ab initio gene predictions. For homology‐based prediction, protein sequences of seven species (*A. thaliana*, *O. sativa*, *S. bicolor*, *B. distachyon*, *H. vulgare*, *Z. mays* and *T. aestivum*) were aligned to the genome assembly using the GeMoMa program (Keilwagen *et al*., 2019) to provide homology‐based evidence. For transcriptome‐based prediction, RNA‐seq data were generated from a diversity of blackgrass tissues collected over developmental time (leaf, main stem, root, developing flowers, mature flowers pre‐anthesis, and mature flowers with pollen). RNA‐seq reads were processed to remove adapters and low‐quality bases and assembled both *de novo* and genome‐guided using Trinity (v.2.4.0) (Haas *et al*., 2013) followed by the Pasa program (http://pasapipeline.github.io) to improve the gene structures. All predicted gene structures were integrated into consensus gene models using the EVidenceModeler (Haas *et al*., 2008). Functional annotation of protein‐coding genes was carried out by comparing alignments to the SwissProt, GenBank nonredundant protein, InterProScan and EggNOG databases. Gene ontology (GO) terms for each gene were obtained from InterPro descriptions. In addition, the gene set was mapped to the Kegg pathway database using BlastKoala (https://www.kegg.jp/blastkoala/) (Kanehisa *et al*., 2016).

### LTR retrotransposons insertion time estimation and expression analysis

As the direct repeat of an LTR‐RT is identical upon insertion, the divergence between the LTR of an individual element reflects the time of the insertion. The insertion date (*T*) for each LTR‐RT was computed by *T* = *K*/2μ, where *K* is the divergence rate and μ is the neutral mutation rate (*K* = −3/4 × log_e_(1−*d* × 4/3), μ = 1.3 × 10^−8^) (Ma & Bennetzen, 2004). Sequence identity (%) between the 5′ and 3′ direct repeats of an LTR candidate is approximated using Blastn, so the proportion of sequence differences is calculated as *d* = 100% − identity%. The tetranscripts package (Jin *et al*., 2015) was used to estimate the expression of LTR‐RTs, and differential expression between samples was analysed in R v.4.0.2 (R Core Team, 2018) using Deseq2 (Love *et al*., 2014).

### Gene duplication and gene family expansion

To identify orthologous and paralogous gene clusters, protein‐coding genes from blackgrass and 11 other species (*A. tauschii*, *T. urartu*, *H. vulgare*, *P. tenuiflora*, *B. distachyon*, *O. sativa*, *Z. mays*, *S. bicolor*, *S. italica*, *E. haploclada* and *A. thaliana*) were analysed using Orthofinder2 (v.2.5.1) (Emms & Kelly, 2015). In cases where there were multiple transcript variants, the longest transcript was selected to represent the gene. A total of 476 single‐copy orthologous genes were identified. Single‐copy genes from each species were aligned using Muscle (Edgar, 2004) and the alignments were concatenated. The concatenated alignment was used to construct a maximum likelihood phylogenetic tree using RAxML (Stamatakis, 2014). The MCMCTree program (Yang & Rannala, 2006) of Paml (Yang, 2007) was used to estimate the divergence time among 12 species. Three calibration time points were used based on previous publications and the TimeTree website (http://www.timetree.org) as normal priors to restrain the age of the node, including 146–154 million years ago (Ma) between Arabidopsis and rice, 68–72 Ma between rice and sorghum, and 49–53 Ma between barley and Brachypodium. Gene family expansion and contraction was determined by comparing the gene cluster size differences between the ancestor and each species with the Café program (De Bie *et al*., 2006). Café uses a model of stochastic birth and death for gene family evolution and a Monte Carlo re‐sampling procedure to calculate the probability (*P*‐value) of a gene family with the observed family size change (expansion or contraction). The threshold for significance was set at *P* ≤ 0.05. To determine the possible whole genome duplication events in the blackgrass genome, we performed a self‐alignment using Last (v.963) (Kielbasa *et al*., 2011) and identified syntenic blocks with MCscanX (Wang *et al*., 2012). For each gene pair within syntenic blocks, synonymous divergence levels (*K*_s_) were calculated using the YN model in the KaKs_Calculator (Wang *et al*., 2010). The *K*_s_ values of all gene pairs were plotted to identify putative whole genome duplication events. To calculate the genome duplication and gene family expansion events, the formula *T* = *K*_s_/2*R* was used, where *R* is the rate of divergence of nuclear genes in plants, which was set to 6.1 × 10^−9^, according to Lynch & Conery (2000).

### QTL‐seq analysis (bulk segregant analysis of SNPs)

Leaf tissue was harvested from unsprayed tillers of all 25 ‘R’ and ‘S’ plants from each F_2_ family. In all cases, young leaf material was collected over 1 h at midday, from each plant into separate 5 ml Eppendorf tubes. Each sample was immediately flash frozen in liquid nitrogen (LN2) and stored at −80°C. Samples were homogenised in LN2 using a micro‐pestle. For bulk segregant analysis, four bulks were made by pooling DNA from all 25 selected individuals from each phenotypic group (herbicide resistant ‘R’, and susceptible ‘S’, in each of the CC2 and CC5 F_2_ families). Illumina paired‐end reads were processed to remove adapters and low‐quality sequences using Trimmomatic (Bolger *et al*., 2014). Cleaned read data were generated after removing reads with > 10% unidentified nucleotides (N), > 30% bases had Phred quality scores < 20 and < 75 bp of read length. Cleaned reads were then mapped to the blackgrass reference genome using Bwa. Variants were called using BCFtools (http://samtools.github.io/bcftools) and filtered using VCFtools (http://vcftools.sourceforge.net). Single nucleotide polymorphisms (SNPs) were subjected to quality control and removed if they met the following criteria: (1) non‐biallelic SNPs, (2) read depth for SNP > 500 or < 5, (3) mapping quality > 40, (4) genotype quality > 100, (5) missing rate < 10% and (6) SNPs within < 20 bp distance from nearby InDels. The QTL‐seq pipeline was used for calculating the SNP‐index, and the ∆SNP‐index was then calculated by subtracting the SNP‐index of one bulk from that of another bulk (Takagi *et al*., 2013).

### RNA‐seq analysis

An RNA‐seq analysis was also conducted using the 25‐herbicide resistant ‘R’ and susceptible ‘S’ plants from each F_2_ family. For each phenotypic group, five replicate RNA‐bulks were created by pooling RNA from five individual plants. RNA was sequenced using standard Illumina TruSeq mRNAseq protocols. The quality of the RNA sequences derived from each sample was assessed using FastQC v.0.11.8 (Andrews, 2010) and pre‐processed as described earlier. The trimmed reads for each sample were mapped to the blackgrass genome using Hisat2 v.2.2.1 (Kim *et al*., 2019) with default parameters except for minimum alignment score parameters of L, 0, −0.6. Reads that mapped to coding sequences of annotated genes were counted using FeatureCounts v.1.6.4 (Liao *et al*., 2014) with default settings. Differential gene expression between samples was analysed in R v.4.0.2 (R Core Team, 2018) using Deseq2 (Love *et al*., 2014).

The expression of all technical replicates was checked before analysis. First, all counts data were transformed using the regularised log‐transform function ‘rlog()’ of the Deseq2 package. Transformed data were visualised using both a principal component analysis and hierarchical clustering of the Euclidean distance between samples. Visual inspection of these results identified one clear outlier sample (CC5 ‘S’ sample A), which was excluded from further analysis. A pre‐filtering step was used to remove genes with zero or low counts before differential expression analysis. First, counts were summed across technical replicates to leave only biological samples. Next, genes were removed if they did not have at least one read per million in at least four samples (where four is equal to the minimum number of reps per treatment level) as per Anders *et al*. (2013). The filtered, biological replicates were analysed using the ‘DESeq()’ function of the Deseq2 package in R, specifying four phenotypic groups: CC2 ‘S’, CC2 ‘R’, CC5 ‘S’ and CC5 ‘R’. In total, 19 937 genes and 19 biological replicate samples were included in this analysis. To generate lists of differentially expressed genes (DEGs), specific comparisons were extracted for the ‘R’ vs ‘S’ samples within each family from this fitted model. Only genes which were significant (*P* < 0.05) and with at least 1.5× fold difference in expression were categorised as differentially expressed. The resultant lists of DEGs for the CC2 and CC5 families were then intersected, to identify DEGs common to both.

Gene ontology information was combined from the SwissProt, EggNOG, and InterPro annotation files to create a single Gene:GO association map, containing 905 051 associations between 28 498 genes and 13 192 GO terms. Gene ontology enrichment analysis was performed for DEGs using TBtools (https://github.com/CJ‐Chen/TBtools). The Gene:GO association map was specified as a custom gene category mapping to use for analysis, and resultant *P*‐values were adjusted using the Benjamini and Hochberg method to control for false discovery rate. In addition, overrepresentation of DEGs on each chromosome was tested using a Fishers' exact test as per Giacomini *et al*. (2020). For each chromosome, the observed number of DEGs was tested against the expected number given chromosome length and number of genes encoded. Resultant *P*‐values were Bonferroni adjusted to account for multiple testing before ascribing significance.

### Gene co‐expression network construction

Trimmed means of *M*‐values were calculated from mapped RNAseq data using the edge‐R package in R (Robinson *et al*., 2010) to construct a gene expression matrix (GEM). The GEM was log_2_ transformed and quantile normalized in R (R Core Team, 2018). The traditional gene co‐expression network (GCN) was created using the Knowledge Independent Network Construction tool (Kinc v.3.4.0) (Shealy *et al*., 2019). A gene correlation matrix was constructed using the Spearman rank correlation coefficient approach (Song *et al*., 2012) with the following Kinc‐specific parameters: ‐‐minsamp 15 –minexp ‐inf –mincorr 0.5 –maxcorr 0.99. A threshold for correlation was determined using the random matrix theory approach with the following parameters: ‐‐tstart 0.95 –tstep 0.001 tstop 0.5 –threads 1 –epsilon 1 e‐6 –mineigens 50 –spline true –minspace 10 –maxpace 40 –bins 60 and was determined to be 0.919. The network was extracted using the extract function and visualized in Cytoscape v.3.9.0 (Shannon *et al*., 2003). The condition‐specific GCN was constructed using the same GEM and Spearman‐ranked correlation coefficient approach in Kinc, but also incorporated a Gaussian mixed model to determine DEG pair clusters that represent condition‐specific sub‐graphs. Low‐powered edges were determined and filtered with the ‘corrpower’ function with an alpha of 0.001 and a power of 0.8. An annotation file was prepared in text format with samples either being annotated as ‘resistant’ or ‘susceptible’ and used to run the ‘cond‐test’. Condition‐specific sub‐graphs were extracted and visualized in Cytoscape v.3.9.0 (Shannon *et al*., 2003).

## Results

### Genome assembly and annotation

**Fig. 1:**
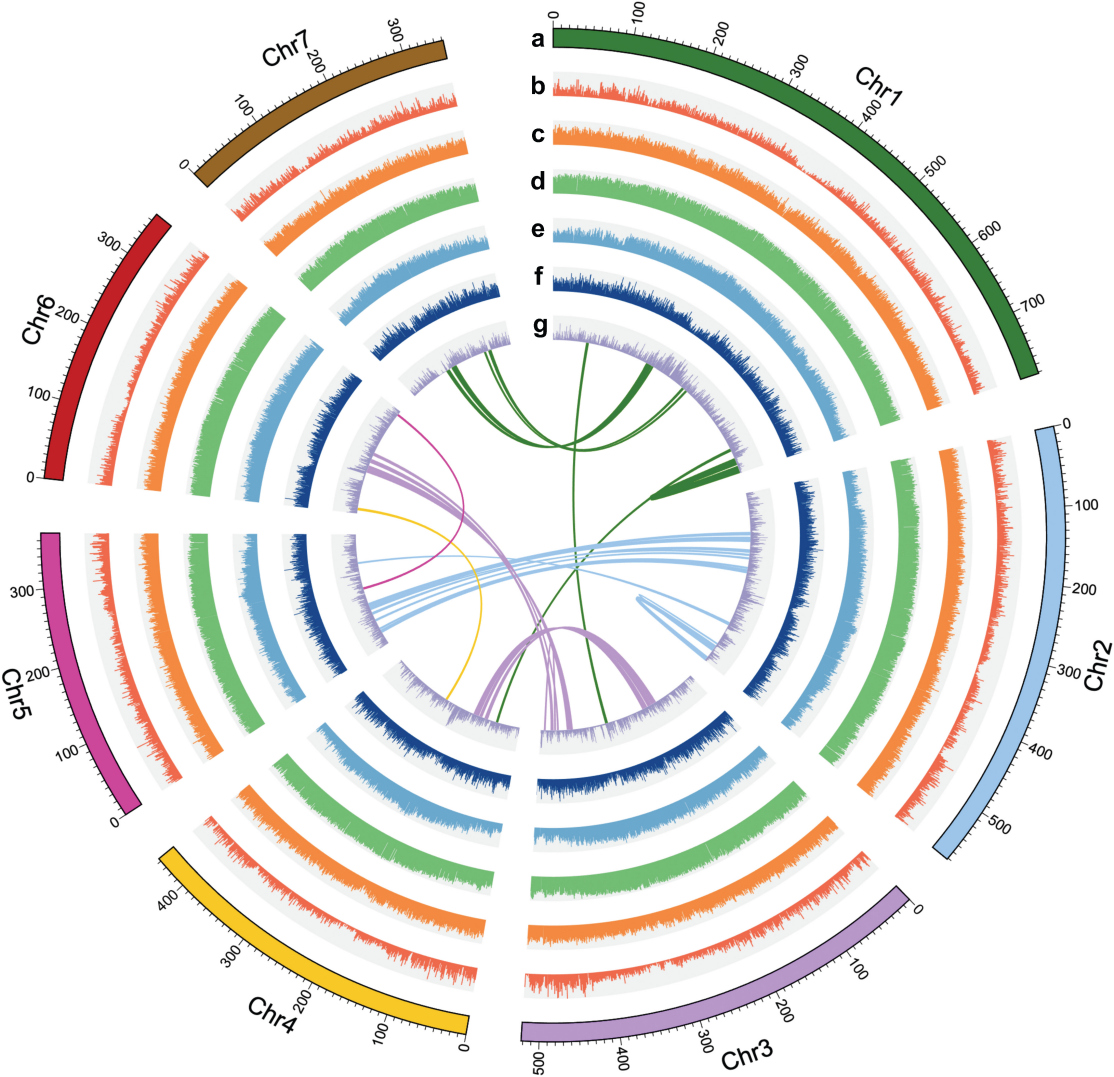
Overview of the *A. myosuroides* genome. A circos graph shows the assembled seven chromosomes (a), distribution of protein‐coding genes (b), distribution of GC content across the genome (c), distribution of transposable elements (d), distribution of *Gypsy* family of long terminal repeats retrotransposons (LTR RTs) (e), distribution of *Copia* family of LTR RTs (f), distribution of SNP/Indel (g). All the histograms (from ‘a’ to ‘g’) were featured in a 1‐Mb sliding window. Connecting line in the centre of the diagram represents a genomic syntenic region covering at least 10 paralogues.

Genome analysis indicated that blackgrass (*A. myosuroides*) has a large genome (3.31–3.55 Gb) and exhibits heterozygosity of 1.52% and repeat content of 84.2% (Tables S1, S2). The high repeat content likely accounts for the large genome size. We adopted a hierarchical sequencing approach that includes complementary single‐molecule sequencing/mapping technologies coupled with deep coverage short read sequences to generate a pseudo‐chromosome reference genome assembly for blackgrass (Fig. S1). The total primary contig length is 3475 Mb, which is consistent with our genome size estimations based on flow cytometry and *k*‐mer analysis (3312–3423 and 3400–3550 Mb, respectively). The final polished blackgrass genome assembly size was 3572 Mb, including 3400 (95.2%) Mb ordered as seven pseudo‐chromosomes with only 172 Mb of unanchored sequences (Fig. 1; Tables 1, S3).

**Table 1:** Assembly statistics of the blackgrass genome.

Characteristics	Values
Assembly size (bp)	3572 044 634
Number of scaffolds	2512
N50 scaffold length (bp)	2255 730
The largest scaffold (bp)	17 744 454
Number of contigs	7866
N50 contig length (bp)	1189 615
The largest contig (bp)	9284 242
GC content (%)	44.66
Total size of pseudomolecules (bp)	3400 051 202
Total size of unanchored sequences	171 993 432
Ns in the assembly	80 915 468
Total size of retrotransposons (bp)	2302 477 515
Total size of DNA transposons (bp)	507 120 408
Total size of repeat sequences (bp)	2851 385 969
Number of genes	45 263
Average length of genes (bp)	2864
Average number of exons per gene	4.3
Total size of genes (bp)	129 639 341
Number of annotated genes	35 999

Both the euchromatic and heterochromatic components of the blackgrass genome are highly complete as supported by Busco scores (96.9% from the *Embryophyta* lineage) (Simao *et al*., 2015) and a LTR assembly index (Ou *et al*., 2018) (LAI: 9.6–35.2, a mean value of 21.9, Table S4; Fig. S2). In addition, the Illumina short reads (81×) returned a 99.6% mapping rate and covered 99.9% of the assembled genome. We identified 8026 403 polymorphisms as SNPs or InDels (Fig. 1g), which corroborates the predicted heterozygosity level of the blackgrass genome. We also observed a high correlation (*r* = 0.98, Table S5) between the assembled chromosome and cytogenic chromosome lengths based on published data (Johnsson, 1944).

We annotated 45 263 protein‐coding genes (mean gene length of 2864 bp) based on *de novo*, homology‐based predictions and transcriptome data from multiple tissues (Fig. S3). Genes were unevenly distributed across the chromosomes with increased gene density towards the distal ends of chromosomes that recedes to low densities in the centre (Fig. 1b). Among these protein‐coding genes, 2385 were annotated as transcription factors. In addition, 4258 non‐coding RNAs were identified, including 1369 transfer RNAs, 941 ribosomal RNAs, 513 micro RNAs and 1425 small nuclear RNAs (Fig. 1 for genome overview).

### Genome dynamics and NTSR

**Fig. 2:**
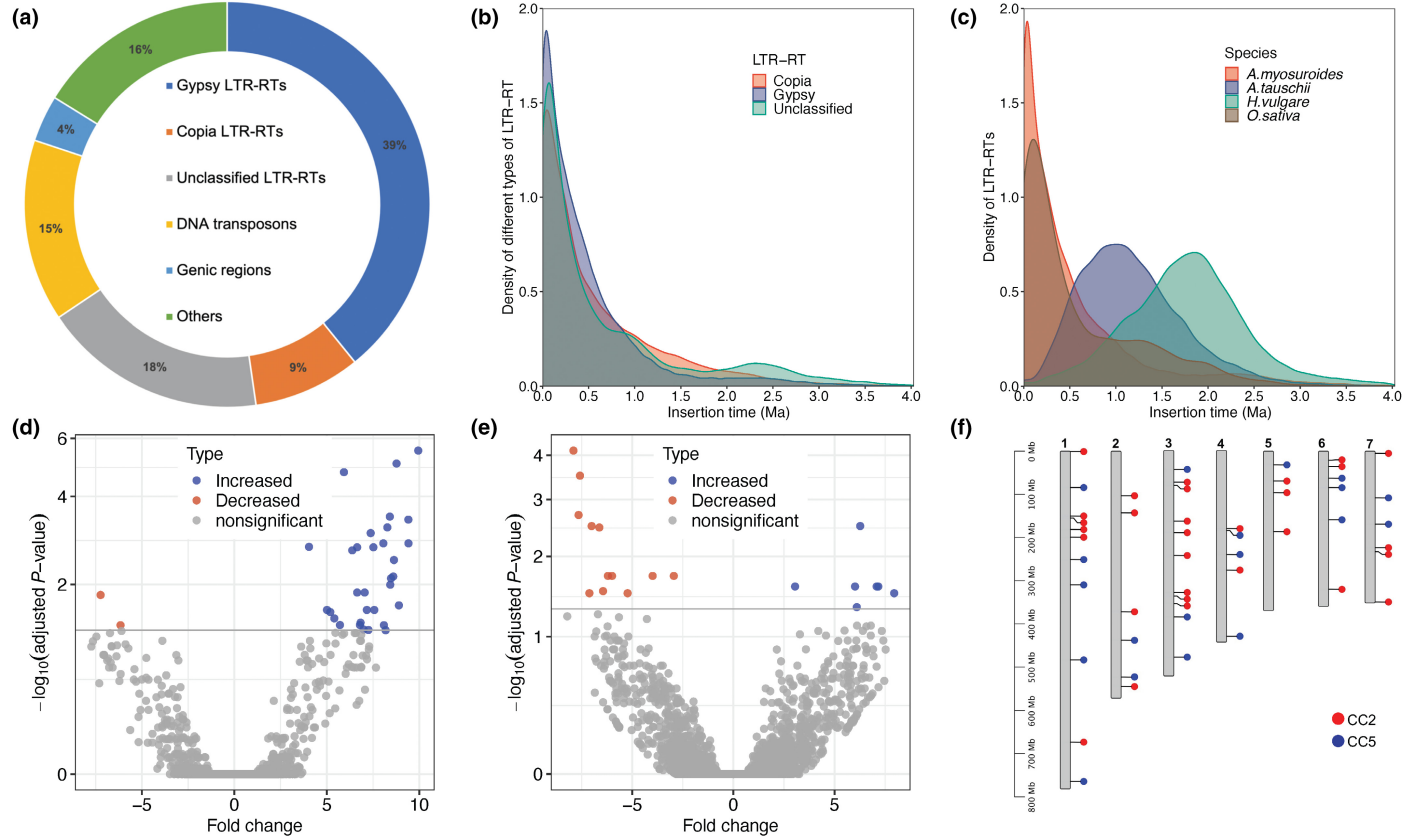
The burst and expression of long terminal repeat retrotransposons (LTR‐RTs) in the *A. myosuroides* genome. (a) Proportions of the major elements in the blackgrass genome, including *Gypsy* LTR‐RTs, *Copia* LTR‐RTs, unclassified LTR‐RTs, DNA transposons, coding DNA and unannotated sequences. (b) The insertion time distribution of different types of LTR‐RT in the blackgrass genome. (c) The insertion time distribution of intact LTR‐RTs in the blackgrass genome compared with those in goatgrass (progenitor of the wheat D genome), barley and rice. (d, e) Volcano plots show differentially expressed LTR‐RTs in CC2 and CC5 family, respectively. Resultant *P*‐values were Bonferroni‐adjusted to account for multiple testing before ascribing significance. (f) The distribution of differentially expressed LTR‐RTs on seven blackgrass chromosomes.

We annotated 2851 Mb (81.7%) of sequence in the assembled genome as TEs (Table S6). The dominant type of TE was LTR retrotransposons (RTs), representing *c*. 80.3% (2290 Mb) of annotated TEs and amounting for 65.6% of the blackgrass genome size (Fig. 1d). *Gypsy*, *Copia* and unclassified RT elements contributed to 39.2%, 8.6% and 17.9% of the genome size, respectively (Fig. 1e,f). DNA transposons contributed to 14.5% of the genome (Fig. 2a). LTR‐RTs are highly unstable and have played an important role in the evolution of plant genomes (Fedoroff, 2000). We observed a single peak of insertion time, occurring *c*. 0.1 Ma, for *Gypsy*, *Copia* and unclassified RTs in blackgrass, which suggests a recent burst of LTR RTs in the genome (Fig. 2b). In addition, we observed a burst of RTs in blackgrass that occurred more recently than those in barley (*Hordeum vulgare*) and goatgrass (*Aegilops tauschii*) but occurred at a similar time in rice (*Oryza sativa*) (Fig. 2c). We observed that LTR‐RTs exhibited different expression profiles between susceptible and resistant plants. In all, 33 and 19 LTR‐RTs exhibited different expression levels between susceptible and resistant plants in CC2 and CC5 families, respectively (Fig. 2d,e). However, common differentially expressed LTR‐RTs were not observed between CC2 and CC5 (Fig. 2f). These results indicate that different LTR‐RTs were historically activated and potentially contribute to NTSR in our different blackgrass populations.

**Fig. 3:**
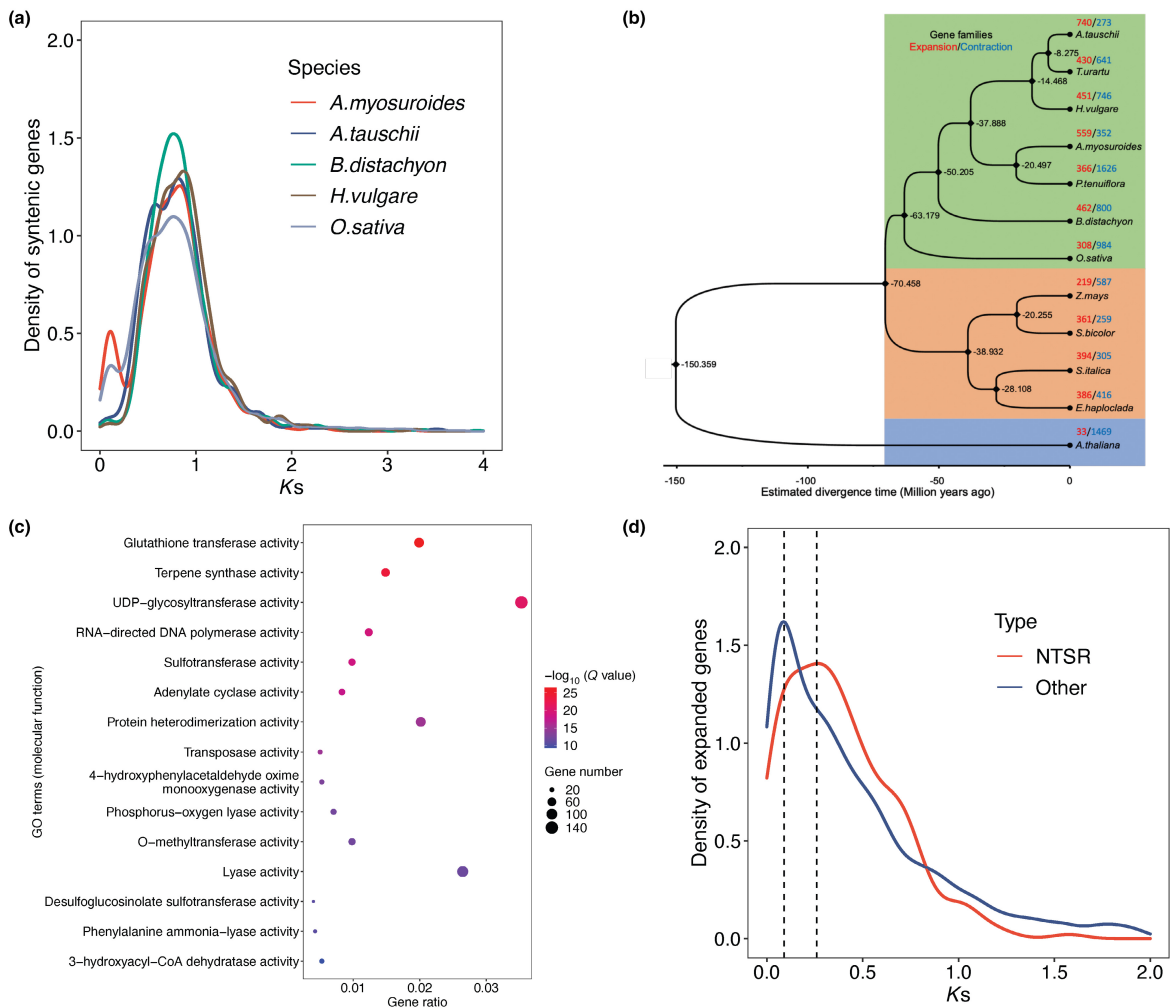
Whole genome duplication and gene expansion in the *A. myosuroides* genome. (a) The frequency distribution of synonymous substitution rates (*K*_s_) of paralogous genes within each plant genome. A shared whole genome duplication event for grasses was assigned to the peak. (b) Phylogenetic tree of 12 plant species and gene family expansion and contraction. Inferred divergence time is denoted at each node in black. Numerical values beside each node show the estimated divergence time of each node. The red and blue numbers above the species name indicate the total number of expanded and contracted gene families, respectively. (c) Gene ontology (GO) enrichment analysis of expanded gene families in the blackgrass genome (molecular function category). The significant levels were Bonferroni‐adjusted to account for multiple testing. (d) The frequency distribution of synonymous substitution rates (*K*_s_) of expanded genes. Expanded genes include those from either NTSR‐related or non‐NTSR (other) gene families.

Genomic duplications, including gene family expansions, can be a result of polyploidization events and signatures of stress adaptations. In blackgrass, we observed two distinct peaks at *K*_s_ values of 0.1 and 0.8 (Fig. 3a). The peak at *c*. 0.8 was shared in all grass species investigated, suggesting blackgrass underwent the same ancient whole genome duplication in the ancestor of *Poaceae* species *c*. 65.6 Ma (Paterson *et al*., 2004). The peak at 0.1 is not apparent in these other species, suggesting that this duplication event is unique to blackgrass. We further examined paralogous gene content within the duplication events and found that the peak at 0.1 (corresponding to 8.2 Ma) was evidenced by a high density of ‘co‐located’ paralogous genes on chromosomes 1, 2 and 3 (Fig. 1), accounting for 10% of total paralogous genes. These results suggest the blackgrass genome underwent small‐scale local duplication events after the occurrence of whole genome duplication. To proximate gene family evolution between blackgrass and other grasses, we constructed a phylogenetic tree based on the concatenated sequence alignment of the 476 single‐copy orthologous genes shared by blackgrass and 11 other species (Fig. 3b). We next examined gene family evolution through expansion and contraction events. A total of 33 757 orthologous gene families composed of 382 550 genes were identified from 12 species, of which 6470 gene families were shared by all the species (Fig. S4). In blackgrass, a total of 559 and 352 gene families were identified with significant expansion and contraction, respectively (*P* <0.05). Gene ontology enrichment analysis of the expanded genes revealed that they were mainly related to multiple enzymatic functions, including glutathione *S*‐transferase (GST), UDP‐glycosyltransferase (UGT) and monooxygenases, all of which have been reported (Gaines *et al*., 2020) to be associated with non‐target site herbicide resistance (Fig. 3c). Here, we define ‘NTSR‐related gene families’ as GST, UGT, cytochrome P450 (P450), ATP‐binding cassette (ABC) transporters, and aldo‐keto reductase (AKR). Using that definition, out of 5440 genes from 559 expanded families, 408 (7.5%) were identified as NTSR‐related genes. The *K*_s_ values for these NTSR‐related gene family expansion events were plotted for all paralog pairs within expanded gene set (Fig. 3d). Peaks at 0.09 (corresponding to 7.3 Ma) and 0.26 (corresponding to 21.7 Ma) were observed for NTSR‐related and non‐NTSR gene families, respectively. Therefore, the expansion of the NTSR‐related gene families greatly predated the use of herbicide, suggesting the possibility that standing genetic variation may have facilitated the rapid evolution of herbicide resistance contributing to weediness in this species.

### QTL‐seq bulk segregant analysis for NTSR

**Fig. 4:**
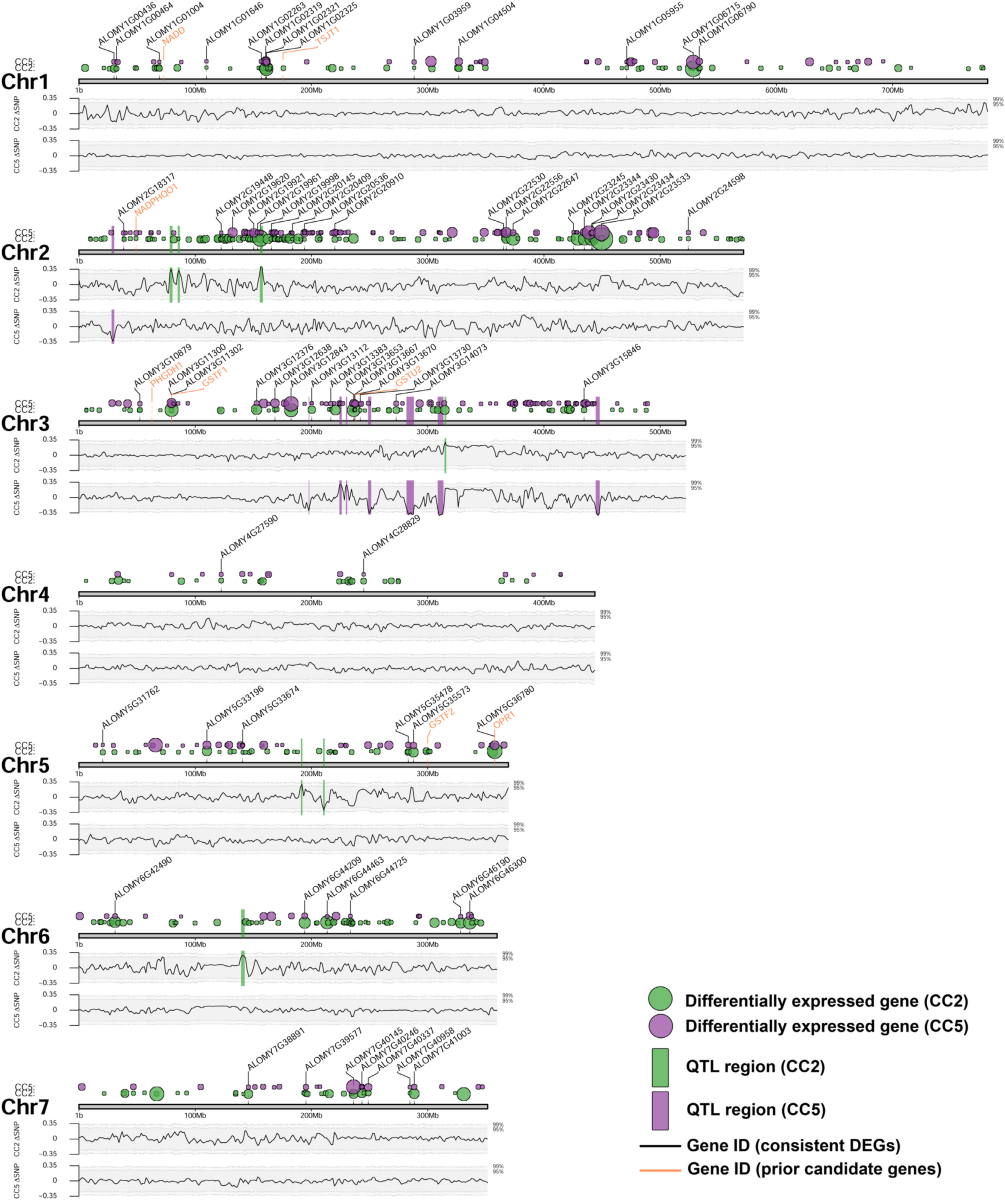
Location across the *A. myosuroides* genome of the differentially expressed genes (DEGs) associated with the NTSR trait. Green and purple circles show the position of DEGs identified in the CC2 and CC5 seed families, respectively. Circle sizes are relative to the adjusted *P*‐value, whereby larger circles denote stronger significance. DEGs consistent across both families are marked with black labels, while orange labels show the position of previously reported NTSR candidate genes. Lower sections show the change in ΔSNP‐index across these chromosomes for the CC2 (top) and CC5 (bottom) families. Shaded regions represent the 95% and 99% confidence bounds for each SNP. Vertical green and purple bars show the quantitative trait loci (QTL) regions for the CC2 and CC5 families, identified from their ΔSNP‐index. *P*‐values were computed based on the Wald test in the Deseq2 package and then adjusted using the Benjamini and Hochberg method for multiple test correction.

To identify the genomic regions controlling herbicide resistance, we performed bulk segregant analysis in the CC2 and CC5 families to identify ΔSNP values with trait significance (Takagi *et al*., 2013; Kumar *et al*., 2020). We obtained 3402 057 and 3205 888 reliable SNPs for each of the CC2 and CC5 families, respectively (Fig. S5). We identified seven significant QTLs in the CC2 family distributed among chromosomes 2, 3, 5 and 6 (Table S7). In the CC5 family, we identified eight QTLs distributed mainly on chromosome 3, with one region on chromosome 2 (Table S7). A total of 371 genes were encoded within the 15 identified QTLs, with each QTL containing between 10 and 58 genes. Interestingly, there was no overlap between QTL regions identified in the two seed families (Fig. 4). Among the 15 identified QTL regions, seven contain genes that are differentially expressed between susceptible and resistant plants of either family; six of them contain transcription factors. The most significant QTL was identified on chromosome 2 in the CC2 family, which covered 2.5 Mb and contains 33 candidate genes. An NADPH‐dependent AKR gene was present in this region which is upregulated in the resistant plants of both CC2 and CC5 families. Members of this gene family have been reported to be associated with herbicide detoxification in other weed species (Pan *et al*., 2019). These results suggest that although single large effect genes (like this AKR gene) could be conferring resistance, it is more likely that NTSR in blackgrass involves multiple independent loci (polygenic trait), and our data provide strong evidence that NTSR mutations are population specific.

### RNA‐seq analysis of NTSR

**Fig. 5:**
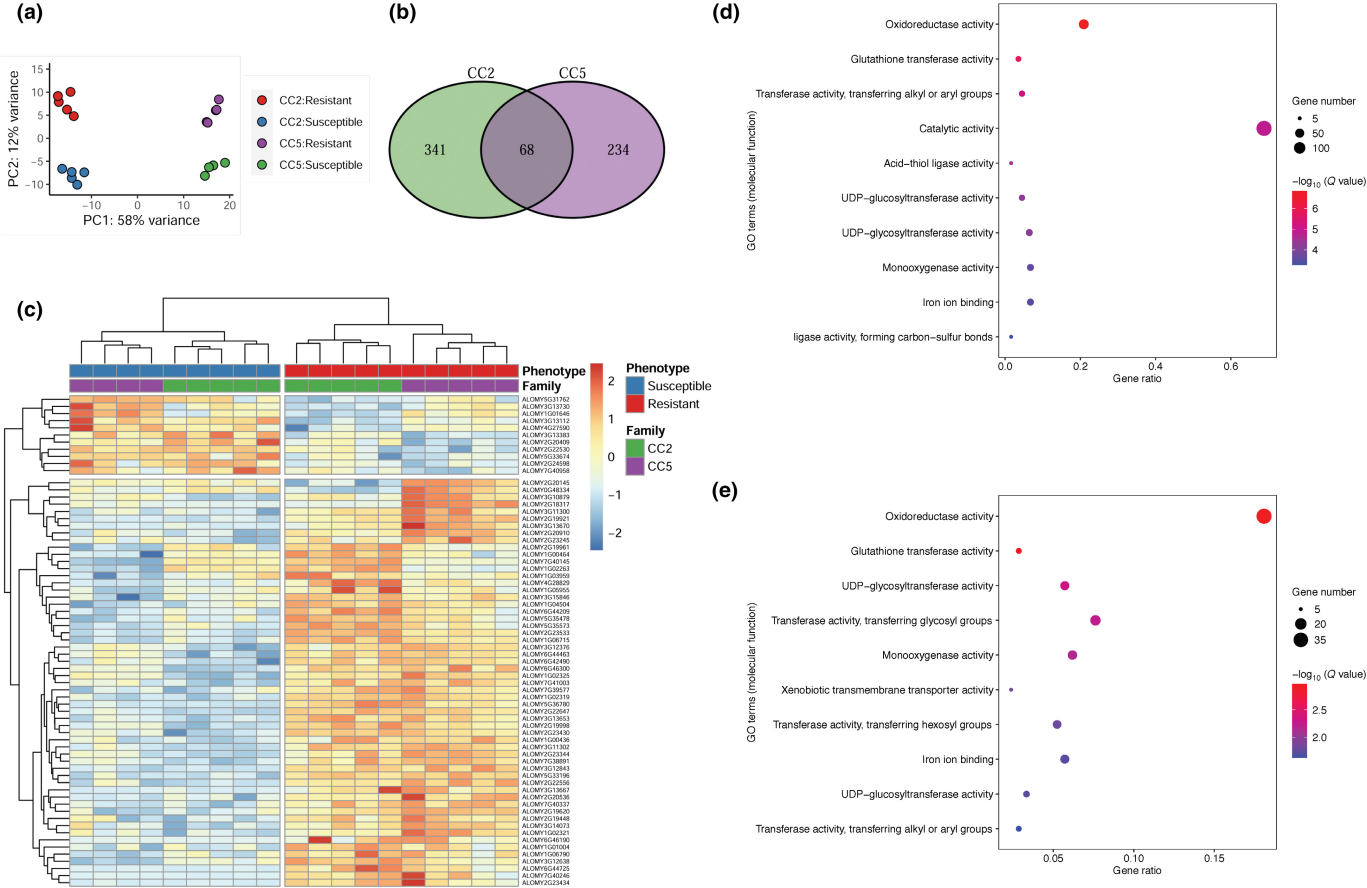
Differential gene expression analysis of the *A. myosuroides* seed families CC2 and CC5, segregating for the NTSR herbicide‐resistant trait. (a) Principal component analysis using all gene expression data. (b) Numbers of differentially expressed genes (DEGs) comparing the ‘R’ (green) and ‘S’ (purple) groups within each family. (c) Heatmap and hierarchical clustering of the 68 DEGs consistently associated with NTSR across both seed families. (d, e) Gene ontology terms, significantly overrepresented in the CC2 and CC5 families, respectively. The significant levels were Bonferroni‐adjusted to account for multiple testing.

To identify DEGs between susceptible and resistant plants, we performed RNA‐seq analysis in the most and least resistant fractions of the two seed families (CC2 and CC5). Principal component analysis of gene expression data (19 937 genes across 19 biological samples) indicates that both seed families and resistance phenotypes contain significant sources of variation between samples (Fig. 5a). Seed family (CC2 vs CC5) was the stronger source of variance accounting for *c*. 58% of the total variance on the first Principal Component (PC1). Within each seed family, the herbicide‐resistant ‘R’ samples form separate clusters from their susceptible ‘S’ counterparts on PC2, with this axis representing 12% of the total variance in gene expression. In each seed family, the ‘direction’ of separation of ‘R’ samples from ‘S’ on PC2 is the same. Principal component analysis of each seed family independently (Fig. S6) revealed that the ‘R’ and ‘S’ samples in each family formed separate clusters on the first principal component (PC1). Within the CC2 and CC5 families, respectively, this PC accounted for 35% and 39% of the total variance in gene expression. These results demonstrate that the presence of the NTSR trait has a considerable impact on constitutive gene expression, even in the absence of herbicide treatment.

Differential expression analysis between ‘R’ and ‘S’ samples across the two seed families identified 643 DEGs (Fig. 5b). A subset of 68 genes were found to be differentially expressed in both seed families. Hierarchical clustering of these 68 genes confirmed that resistance phenotype was a greater source of variability than seed family, and 81% (55) of these 68 genes were upregulated in ‘R’ samples relative to ‘S’ for both families (Fig. 5c). Within the 68 consistent DEGs, we found three of eight previously recorded blackgrass NTSR candidate genes: *AmGSTF1*, *AmGSTU2* and *AmOPR1* (Cummins *et al*., 2013; Tetard‐Jones *et al*., 2018). In each case, the expression of these three candidate genes was significantly higher in the ‘R’ phenotype than the ‘S’ (Fig. S7), agreeing with previously reported findings. In addition, three additional significant genes, ALOMY2G19998 (paralog of AKR4‐1 in *Echinochloa colona*), ALOMY1G02321 (paralog of CYP81A10v7 in *Lolium rigidum*) and ALOMY6G42490 (paralog of ABCC8 in *Echinochloa colona*), were all upregulated in both CC2 and CC5 families (Fig. S7). The orthologs of these three NTSR‐related genes have been validated to endow NTSR to various herbicides in other weed species (Pan *et al*., 2019, 2021; Han *et al*., 2021). These findings further reinforce the potential importance of these six genes, having now been implicated in the regulation of the herbicide metabolism phenotype across multiple populations, multiple independent studies and even multiple species.

Nevertheless, although 68 DEGs overlapped between the two families, the majority (341 for the CC2 family and 234 for the CC5 family) were unique to one family or the other (Fig. 5b). Differential expression associated with herbicide resistance was observed for another 12 P450s and five GSTs within the CC2 family, while five P450 genes displayed differential expression unique to the CC5 family. Two ABC transporters were identified as significant; one differentially expressed in the CC2 family, the other within the CC5 family. Comparably, several genes within the glycosyltransferase, drug/metabolite transporter and disease‐resistant NB‐LRR families were shown to have differential expression unique to one family or the other. Separate gene set enrichment analysis of DEGs for each family identified both shared and unique GO terms. Most of the shared overrepresented GO terms have been reported to be associated with NTSR, including GST, UGT and some cytochrome P450 superfamilies. ‘Xenobiotic transmembrane transporter’ activity was only overrepresented in CC5 (Fig. 5d,e), indicating the possible family‐specific mechanism of resistance for CC5. These results add to the growing evidence supporting a role for these gene families in herbicide detoxification, while the extent of DEGs specific to each family implies that different NTSR blackgrass populations may acquire an individual ‘profile’ of DEGs from these gene families.

In addition to previously reported genes and gene families, we found two transcription factors (ALOMY1G01646 and ALOMY2G19620), which were differentially expressed in both families, and the corresponding GO term (GO:0042221, ‘response to chemical’) was significantly enriched in both CC2 (*P* = 0.016) and CC5 (*P* = 0.006). A further nine and seven transcription factors with altered expression were identified in the CC2 and CC5 family, respectively. Although not a causal link, this may represent some involvement of these genes in regulation of NTSR gene expression. Interestingly, two Acetyl‐CoA synthetase‐like ATP‐dependent AMP‐binding enzymes were consistently upregulated in resistant plants: ALOMY2G20910 and ALOMY6G44209. Their corresponding GO term (GO:0032787, ‘monocarboxylic acid metabolic process’) was also significantly enriched in both CC2 (*P* = 0.006) and CC5 (*P* = 0.031). AMP‐forming acetyl‐CoA synthetases (ACS) catalyse the formation of acetyl‐CoA, substrate for the ACCase enzyme which is the target for herbicidal inhibition. Altered expression of these two genes could signify some remodelling of this pathway upstream of the point of herbicidal inhibition.

### Co‐localisation of NTSR‐related features (QTLs and DEGs)

In addition to the population‐specific profile of DEGs, no overlap was observed between QTL regions identified in the two seed families (Table S7). However, 12 of the 15 total QTL regions were located on chromosomes 2 and 3 (Fig. 4). These two chromosomes also showed the greatest density of DEGs identified in the RNA‐seq analysis, with almost half (33) of the 68 consistent DEGs located on these two chromosomes, along with half of the previously reported NTSR candidate loci for this species (Fig. 4). In total, chromosomes 2 and 3 combined accounted for 45% and 55% of the total DEGs observed in the CC2 and CC5 families, respectively. Results of a Fishers' exact test for overrepresentation of DEGs per chromosome confirmed that chromosome 2 (CC2 family) and chromosomes 2 and 3 (CC5 family) were significantly enriched in resistance‐associated DEGs (Table S8). These results suggest that chromosomes 2 and 3 are ‘hot‐spots’ for NTSR evolution in this species.

**Fig. 6:**
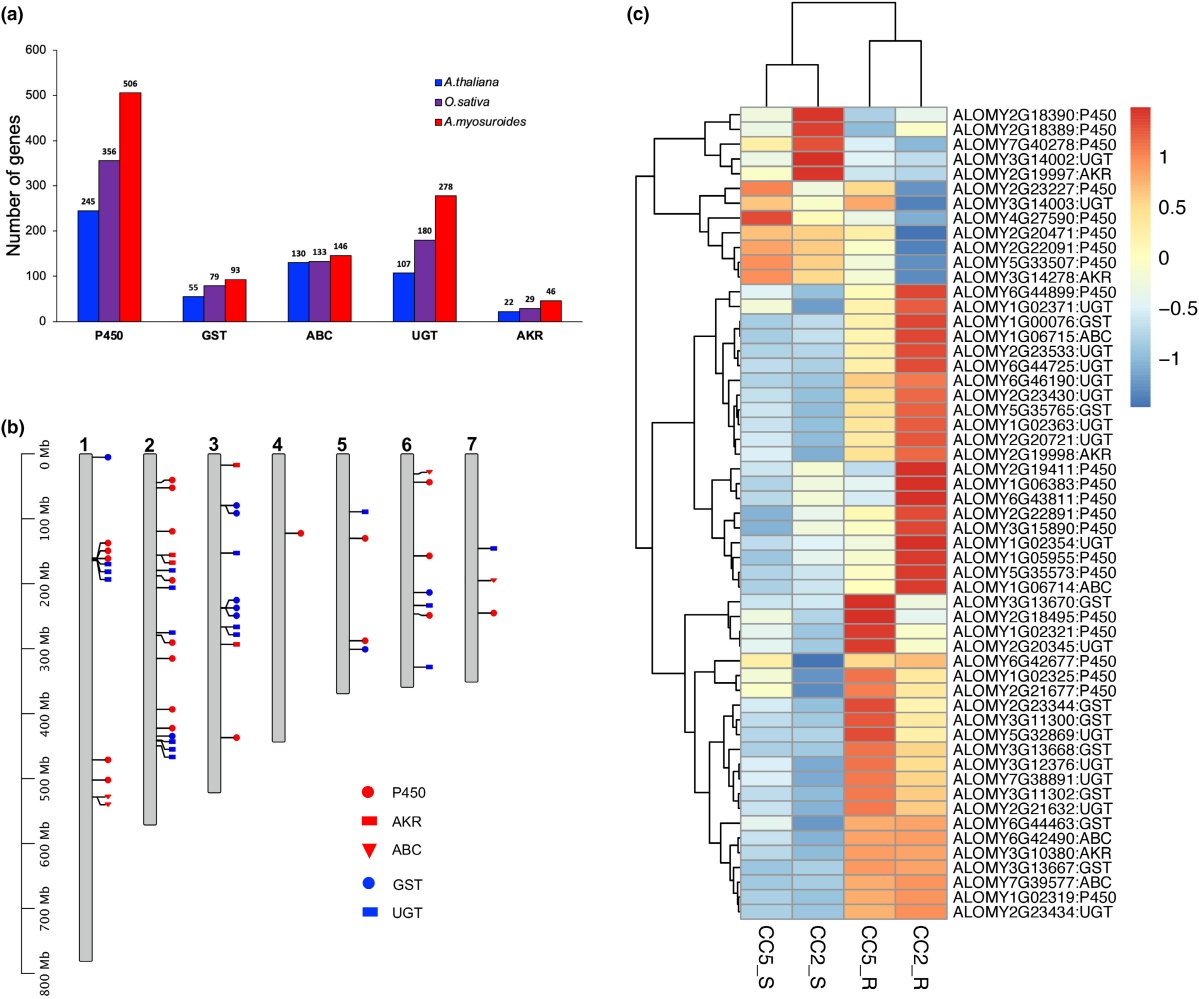
The gene number, distribution and expression for five non‐target site resistance (NTSR)‐related gene families in the *A. myosuroides* genome. (a) The number of genes identified for five NTSR‐related gene families including cytochrome P450 (P450), glutathione *S*‐transferase (GST), ATP‐binding cassette transporters (ABC), UDP‐glycosyltransferases (UGT) and aldo‐keto reductase (AKR). (b) The distribution of differentially expressed genes (DEGs) from five NTSR‐related gene families on seven blackgrass chromosomes. (c) Heatmap of the DEGs from five NTSR‐related gene families.

To examine this further, we performed a comprehensive genome‐wide analysis of the five principal NTSR‐related gene families (GST, UGT, P450, ABC and AKR) (Fig. 6). A total of 506, 93, 146, 278 and 45 genes were identified in the blackgrass genome for P450, GST, ABC, UGT and AKR, respectively. Overall, blackgrass has a larger proportion of NTSR‐related genes (1069, 2.48% of total gene number) in the genome compared with those in *Arabidopsis* (2.04%) and rice (2.18%) genomes (Table S9; Fig. 6a). Among the five NTSR‐related gene families, blackgrass only has a smaller proportion for ABC family compared with *Arabidopsis* and rice. This observation is in line with the gene family expansion analysis, where P450, UGT and GST families each contain a high ratio of expanded genes. For example, 180 (35.6%), 61 (65.6%) and 129 (46.4%) genes were expanded in P450, GST and UGT gene families, respectively. However, only 50 and 28 NTSR‐related genes were differentially expressed between susceptible and resistant plants in CC2 and CC5, respectively. A large proportion (50%) of these differentially expressed NTSR‐related genes were located across chromosomes 2 and 3 combined in both seed families (Table S10; Fig. 6b). Nevertheless, tests for overrepresentation were non‐significant, in part due to the number of total NTSR‐related genes also being high across these two chromosomes (416 of 1068 NTSR‐related genes). Most of the differentially expressed NTSR‐related genes were upregulated in resistant plants and 23 of them were shared between families, including 6, 6, 3, 7 and 1 shared genes for P450, GST, ABC, UGT and AKR, respectively (Fig. 6c). Predominantly, these results highlight that while genomic features associated with NTSR (QTLs and DEGs) are largely population specific, their significant co‐localisation on chromosomes 2 and 3 reflects the importance of these two chromosomes during NTSR evolution.

### Genetic coordination of NTSR via GCN analysis

**Fig. 7:**
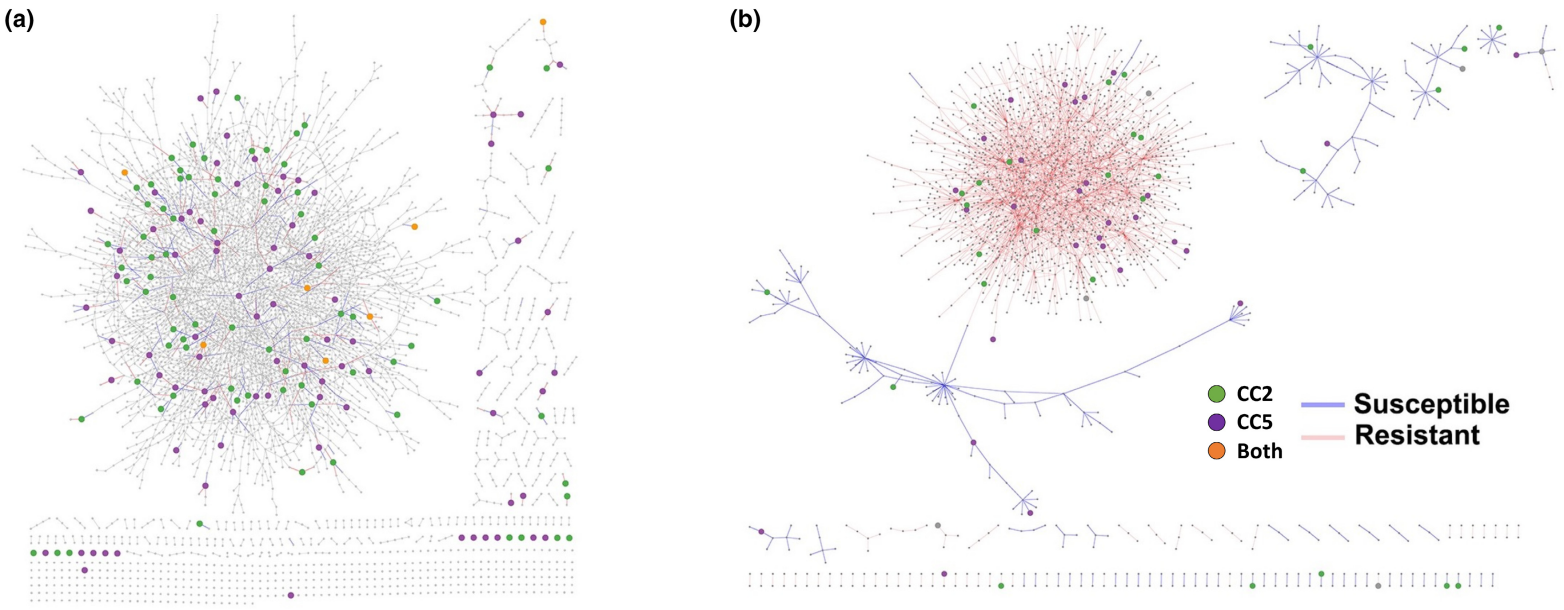
Genetic coordination of non‐target site resistance (NTSR) in CC2 and CC5 families of *A. myosuroides*. (a) Traditional Spearman‐ranked gene co‐expression network derived from RNA‐seq expression that depicts common and unique genetic architecture underpinning NTSR in both the CC2 and CC5 families. Green nodes are unique to CC2, purple nodes are unique to CC5 and orange are common between both families. The graph was filtered for nodes with at least two connections. (b) Condition‐specific gene co‐expression network derived from the RNA‐seq data taking into consideration plant phenotype (herbicide susceptible/resistant).

Gene co‐expression networks were constructed using traditional Spearman‐ranked and condition‐specific approaches that enable alternate strategies to examine the genetic coordination of NTSR mechanisms (Fig. 7a,b, respectively). The traditional Spearman‐ranked coefficient approaches resulted in a total of 16 601 nodes connected by 16 130 edges (Fig. 7a). Hub gene sub‐graphs display significant co‐expressed gene interaction pairs that include candidate genes from the bulk segregant and RNA‐seq studies. We identified a total of 13 CC2 specific sub‐graphs and 20 for CC5 (Fig. S8a–d). In CC2, we found the NTSR‐related gene families identified in the QTL‐seq analysis, such as GST, AKR and beta‐keto acyl synthase co‐expressed with various transcription factors and other genes that could be involved in their regulation (Fig. S8a,b). An HMG transcriptional regulator is also positively correlated with two genes involved in metabolism: tubulin/FtsZ family gene and a ubiquitin carboxyl‐terminal hydrolase, and negatively correlated with an α‐*N*‐acetylglucosaminidase (Fig. S8e). In the CC5 family sub‐graphs, we identified alternate active genetic machinery that are co‐expressed with genes identified in the QTL regions, such as cytochrome p450s, thioesterase, glycosyl hydrolase, pectinesterase, exostensin gene family and others connected with various classes of transporters and transcription factors/regulators (Fig. S8c,d). The condition‐specific network also partitioned clusters of co‐expressed gene interactions pairs in both a family specific and overlapping manner (Fig. 7b). For example, this approach also identified an AKR and protein tyrosine/serine/threonine kinase unique to CC2. Oxioreductase, peroxidase and vacuolar sorting were among CC5‐specific clusters (Fig. S8f). This approach also identified a largely connected subgraph of connected genes discovered in both CC2 and CC5 bulk‐segregant and RNA‐seq analysis (Fig. S8g). These network analyses further highlight that NTSR in blackgrass is likely to be due to a combination of core and population‐specific loci that act in concert with one another.

## Discussion

Despite the global impacts of weedy plants, few genomic resources have been developed for weed species (see Sharma *et al*., 2021). Here, we present a reference‐grade genome assembly for Europe's most devastating agricultural weed, *A. myosuroides* (Hicks *et al*., 2018; Varah *et al*., 2020), and demonstrate that (1) NTSR in blackgrass is a complex, polygenic trait that evolves from standing genetic variation within stress‐ and defence‐related gene families and (2) that the convergent evolution of the NTSR phenotype among two field‐evolved populations has a notably different genetic basis among populations.

F_2_ seed families were produced for two field‐evolved blackgrass populations with very similar phenotypic resistance to the ACCase inhibiting herbicides and global, constitutive transcript profiles were compared for bulked resistant and susceptible individuals derived from the two seed families. At the transcriptional level, 11% of the resistance‐associated DEGs were common to both seed families. These 68 common DEGs include several genes and gene families previously implicated in NTSR in blackgrass (Cummins *et al*., 2013; Tetard‐Jones *et al*., 2018) and in a range of other weed species with evolved NTSR (Yang *et al*., 2018; Fang *et al*., 2019; Davies *et al*., 2020; Wang *et al*., 2020; Suzukawa *et al*., 2021; Torra *et al*., 2021). There were also commonalities in the localization of genomic signatures for NTSR across the two populations. For example, we found that chromosomes 2 and 3 were significantly enriched in both DEGs and QTL regions associated with resistance in both tested families. These results concur with those of Giacomini *et al*. (2020), who found physical clustering of DEGs in *Amaranthus tuberculatus*. Together, these results highlight that there are commonalities observed in the metabolic basis and broad genomic localisation of features associated with independently evolved NTSR.

Our results are consistent with a growing body of evidence from studies that explore the transcriptomic basis of NTSR. These studies confirm that NTSR is conferred by the upregulation of individual genes that are members of large stress‐ and defence‐related gene families; the cytochrome P450 monooxygenases, GSTs, ABC transporters, AKRs, glucosyl transferases and others (e.g. Cummins *et al*., 2013; Pan *et al*., 2019, 2021; Dimaano & Iwakami, 2021; Huang *et al*., 2021). Mounting evidence shows that these gene families are regularly implicated, and common genes were upregulated in both NTSR phenotypes studied here, and in NTSR populations of other species (Pan *et al*., 2019, 2021; Han *et al*., 2021). Together, these studies highlight a notable degree of parallelism in the metabolic NTSR phenotype. This would appear to indicate some constraint in the possible pathways via which NTSR can evolve. It is important to note, however, that the majority of DEGs identified in our study were not common among the two seed families; 89% were specific to one family or the other. This suggests that though a core of commonly over‐expressed genes is key for the metabolic expression of NTSR among populations, a significant number of population‐specific genes also contribute, indicating that both parallel and non‐parallel changes occur at the level of plant metabolism associated with the independent evolution of NTSR. Further studies that explore associated functional alleles in a greater number of evolved populations are warranted, and will be required to more completely understand the relative importance of parallelism and non‐parallelism in the evolution of the NTSR transcriptome.

Although the metabolic basis of NTSR among populations provides evidence for parallelism, our results unequivocally indicate that there is a discrete, non‐parallel basis to NTSR at the genomic level. Of the 15 significant QTLs identified (8 and 7 in the two seed families, respectively), there were no overlapping QTL regions, though significant QTLs were over‐represented on chromosomes 2 and 3 in each population. These observations suggest that while selection for NTSR may be localized to certain regions of the genome, the genetic basis of these traits is quite different among blackgrass populations. These results are consistent with the conclusion in Van Etten *et al*. (2020) and Gupta *et al*. (2021), that NTSR in *Ipomoea purpurea* is conferred by multiple loci. Similarly, separate studies of HPPD resistance in different *Amaranthus tuberculatus* populations have highlighted the distal regions of the genome showing signatures of selection for resistance (Kohlhase *et al*., 2018; Murphy *et al*., 2021). Our findings, combined with our co‐expression network analysis, provide strong evidence that NTSR among blackgrass populations is divergent at the genomic level.

Assessment of repetitive genome structure and duplication arrays suggests that these elements themselves might serve as an underlying mechanism facilitating rapid adaptation in blackgrass. For instance, high heterozygosity, expanded gene families (Fig. 1), a relatively recent (and unique) genome duplication event (Fig. 2), and clusters of TEs and LTR‐RTs are signatures of a dynamic genome. It is notable that the paralogous genes associated with genome duplication in this species are located on chromosomes 1, 2 and 3, among the densest regions of significant QTLs and DEGs (Fig. 4). We speculate that high levels of variation in the number, regulation and function of these defence‐related gene families enable weedy species such as blackgrass to rapidly evolve NTSR via exaptation of genes within these large multi‐functional gene families. Variation within these gene families distributed among discrete genetic backgrounds of blackgrass likely underpin the potential for non‐parallel evolution of NTSR.

### Conclusion

We have established that NTSR in blackgrass is a polygenic trait, and that the genetic basis of NTSR can be markedly different between independently evolved populations; albeit underpinned by the upregulation of some common metabolic pathways. Notably, we find evidence for multiple QTL associated with the NTSR phenotype, but no evidence that these QTLs are the same among independently evolved populations. On this basis, we conclude that the landscape‐scale evolution of NTSR results from both parallel and non‐parallel patterns of evolution across the genome, as reported by Van Etten *et al*. (2020) and Kreiner *et al*. (2021). These findings have wide significance for understanding the potential for rapid plant adaptation under novel and changing environments. They suggest that large and plastic plant genomes harbour sufficient standing genetic variation to enable rapid adaptation to novel stresses. The associated duplication and redundancy in plant genomes means that adaptation may not be mutation limited and that the repeated evolution of resistance and/or tolerance relies on neither rare mutational events nor hard selective sweeps. They also hint that complex adaptations to abiotic and biotic stresses are not constrained by genetic variation and architecture and that convergent phenotypes are shaped by population‐specific genome structure and plasticity.

## Competing interests

None declared.

## Author contributions

PN, CS and RB conceived the study and assembled project funding. CL, DC and PN provided characterised plant material for sequencing. LC and CS assembled and annotated the blackgrass genome. LC, DC and CS analysed genomics and transcriptomics datasets and PN, DM and RB contributed to discussion and interpretation of data. LC, DC, CS and PN wrote the first draft of the paper and all authors contributed to subsequent editing and improvement. LC and DC contributed equally to this work. RB, PN and CS senior authors contributed equally to this work.

## Supporting information


**Fig. S1** Pipeline of genome assembly for the blackgrass.
**Fig. S2** LAI values across seven assembled blackgrass chromosomes.
**Fig. S3** Pipeline of gene annotation for the blackgrass.
**Fig. S4** The distribution of different types of gene family.
**Fig. S5** SNP marker density for CC2 and CC5 population, respectively.
**Fig. S6** Principal component analysis of all gene expression data.
**Fig. S7** Differential expression of previously reported NTSR candidate genes.
**Fig. S8** Parallel and overlapping sub‐graphs in CC2 and CC5.
**Notes S1** The development of research materials and genome sequencing and assembling.
**Table S1** Genome size estimation based on flow cytometry.
**Table S2** Genome size estimation based on *k*‐mer analysis.
**Table S3** Statistics of the assembled seven chromosomes of *A. myosuroides*.
**Table S4** Busco analysis of genome completeness.
**Table S5** Summary of cytogenic and assembly length of each blackgrass chromosome.
**Table S6** Statistics of the annotated transposon elements.
**Table S7** Summary of identified QTLs from CC2 and CC5 populations.
**Table S8** Statistical assessment of over‐representation of differentially expressed genes.
**Table S9** Summary of gene number for NTSR‐related gene families.
**Table S10** Summary of differentially expressed NTSR‐related gene number.
Please note: Wiley is not responsible for the content or functionality of any Supporting Information supplied by the authors. Any queries (other than missing material) should be directed to the *New Phytologist* Central Office.

## Data Availability

This Whole Genome Shotgun project has been deposited at DDBJ/ENA/GenBank under the accession no. JAPCYS000000000. The version described in this paper is version JAPCYS010000000. Raw genome and transcriptome sequencing reads have been deposited in the National Center for Biotechnology Information BioProject database under the accession no. PRJNA889547. Code availability: the code for RNA‐seq and QTL‐seq analyses is freely available at GitHub (https://github.com/Lichuncai/Blackgrass‐Genome‐Project).
