# Supplementary material for: The blackgrass genome reveals patterns of non‐parallel evolution of polygenic herbicide resistance
Source: New Phytol. 2023 Jan 12;237(5):1891–907. doi: 10.1111/nph.18655 (PMC10108218; doi:10.1111/nph.18655)
Supplement: Supplementary file 1 — Fig. S1 Pipeline of genome assembly for the blackgrass. Fig. S2 LAI values across seven assembled blackgrass chromosomes. Fig. S3 Pipeline of gene annotation for the blackgrass. Fig. S4 The distribution of different types of gene family. Fig. S5 SNP marker density for CC2 and CC5 population, respectively. Fig. S6 Principal component analysis of all gene expression data. Fig. S7 Differential expression of previously reported NTSR candidate genes. Fig. S8 Parallel and overlapping sub‐graphs in CC2 and CC5. Notes S1 The development of research materials and genome sequencing and assembling. Table S1 Genome size estimation based on flow cytometry. Table S2 Genome size estimation based on k‐mer analysis. Table S3 Statistics of the assembled seven chromosomes of A. myosuroides. Table S4 Busco analysis of genome completeness. Table S5 Summary of cytogenic and assembly length of each blackgrass chromosome. Table S6 Statistics of the annotated transposon elements. Table S7 Summary of identified QTLs from CC2 and CC5 populations. Table S8 Statistical assessment of over‐representation of differentially expressed genes. Table S9 Summary of gene number for NTSR‐related gene families. Table S10 Summary of differentially expressed NTSR‐related gene number. Please note: Wiley is not responsible for the content or functionality of any Supporting Information supplied by the authors. Any queries (other than missing material) should be directed to the New Phytologist Central Office. [file NPH-237-1891-s001.pdf]

New Phytologist Supporting Information

Article title: The blackgrass genome reveals patterns of non-parallel evolution of polygenic herbicide resistance

Authors: Lichun Cai, David Comont, Dana MacGregor, Claudia Lowe, Roland Beffa, Paul Neve, Christopher Saski

Article acceptance date: November 23, 2022

Table S1. Genome size estimation based on flow cytometry.

Sample ID	Population	Phenotype	DNA content (pg/2C)	Mbp/2C	Gbp/1C
BG1	Rothamsted	S	6.90	6750	3.37
BG2	Rothamsted	S	6.74	6595	3.30
BG3	Rothamsted	S	6.81	6656	3.33
BG4	PELD	R	6.77	6625	3.31
BG5	PELD	R	7.54	7373	3.69
BG6	PELD	R	6.77	6625	3.31
BG7	LOLA 81	S	6.84	6687	3.34
BG8	LOLA 81	S	6.74	6595	3.30
BG9	LOLA 81	S	7.00	6846	3.42
BG10	LOLA 91	R	6.90	6750	3.37
BG11	LOLA 91	R	6.77	6625	3.31
BG12	LOLA 91	R	6.87	6718	3.36
Average	NA	NA	6.89	6737	3.37

Table S2. Genome size estimation based on k-mer analysis.

k-mer length	Total number of *k*-mer	*k*-mer coverage	Estimated genome size
17	237,986,849,712	70	3400Mb
19	234,416,794,205	68	3447Mb
21	230,846,540,235	66	3498Mb
31	212,995,740,384	60	3550Mb

Table S3. Statistics of the assembled seven chromosomes of *A. myosuroides*.

Chromosome	Anchored scaffold number	Length of chromosome
Chr1	627	781,714,917
Chr2	416	571,710,514
Chr3	350	522,029,352
Chr4	321	443,909,942
Chr5	278	369,436,349
Chr6	264	359,679,614
Chr7	238	351,570,514
Total	2494	3,400,051,202

Table S4. BUSCO analysis of genome completeness.

Description	Gene number	Percentage (%)
Complete BUSCOs (C)	1563	96.9
Complete and single-copy BUSCOs (S)	1497	92.8
Complete and duplicated BUSCOs (D)	66	4.1
Fragmented BUSCOs (F)	14	0.9
Missing BUSCOs (M)	37	2.2
Total BUSCO groups searched	1614	100

Table S5. Summary of cytogenic and assembly length of each blackgrass chromosome.

Chromosome	Cytogenic length (μm)	Assembly length (bp)
1	8.4	781,714,917
2	6.4	571,710,514
3	5.4	522,029,352
4	5.0	443,909,942
5	4.4	369,436,349
6	3.8	359,679,614
7	3.0	351,570,514

Table S6. Statistics of the annotated transposon elements (TEs).

Class	Superfamily	Number	Total size (bp)	Percentage (%)
Class 1	LTR-retrotransposons			
	Gypsy (RLG)	1922808	1,367,269,327	39.16
	Copia (RLC)	445775	298,678,755	8.56
	Unclassified (RLX)	1574609	623,678,411	17.86
	Non-LTR retrotransposons			
	Long interspersed nuclear elements (RIX)	20527	12,053,133	0.35
	Short interspersed nuclear elements (SIX)	2807	797,889	0.02
Class 2	DNA transposons			
	hAT (DTA)	41471	15,852,349	0.45
	CACTA (DTC)	335408	169,435,014	4.85
	Harbinger (DTH)	124608	61,410,666	1.76
	Mutator (DTM)	181317	79,309,130	2.27
	Mariner (DTT)	189514	60,919,894	1.74
	Unclassified (DXX)	24210	11,107,295	0.32
	Helitron (DHH)	248002	109,086,060	3.12
Others	XXX	176175	41,788,046	1.20
Total	TEs	5287231	2,851,385,969	81.68

Table S7. Summary of identified QTLs from CC2 and CC5 populations.

Population	Chromosome	Name	Start(bp)	End(bp)	Length(bp)	Peak delta SNP index	Peak position (bp)	Average delta SNP index
CC2	Chr2	*qtl-cc2-2-1*	78,155,610	80,414,345	2,258,735	0.35	79,286,939	0.31
	Chr2	*qtl-cc2-2-2*	84,968,723	86,789,597	1,820,874	0.33	85,985,573	0.31
	Chr2	*qtl-cc2-2-3*	155,616,708	158,148,961	2,532,253	0.41	156,325,090	0.39
	Chr3	*qtl-cc2-3-1*	314,531,189	315,950,139	1,418,950	0.30	315,049,343	0.27
	Chr5	*qtl-cc2-5-1*	190,968,113	192,166,507	1,198,394	0.29	191,929,219	0.27
	Chr5	*qtl-cc2-5-2*	210,023,549	211,392,868	1,369,319	-0.29	210,693,320	-0.26
	Chr6	*qtl-cc2-6-1*	139,536,063	142,546,311	3,010,248	0.28	140,494,464	0.27
CC5	Chr2	*qtl-cc5-2-1*	28,124,368	30,262,000	2,137,632	-0.33	29,036,282	-0.30
	Chr3	*qtl-cc5-3-1*	197,701,391	198,108,389	406,998	-0.30	197,792,990	-0.27
	Chr3	*qtl-cc5-3-2*	224,118,449	226,070,109	1,951,660	0.30	224,317,480	0.29
	Chr3	*qtl-cc5-3-3*	229,745,161	230,705,090	959,929	0.29	230,421,617	0.28
	Chr3	*qtl-cc5-3-4*	248,724,538	251,387,988	2,663,450	-0.35	249,793,371	-0.33
	Chr3	*qtl-cc5-3-5*	281,782,841	288,136,846	6,354,005	-0.39	283,456,062	-0.35
	Chr3	*qtl-cc5-3-6*	308,719,007	313,553,107	4,834,100	-0.41	309,948,215	-0.37
	Chr3	*qtl-cc5-3-7*	444,582,369	447,919,982	3,337,613	-0.41	446,569,526	-0.37

Table S8. Statistical assessment of over-representation of differentially expressed genes (DEGs) per chromosome, calculated using a one-sided Fishers exact test. Rows in bold represent chromosomes with a significantly greater number of DEGs than expected.

Chromosome	Total	Observed DEG	Expected DEG	Odds ratio	Pval	p.adjust
CC2 seed family						
Chr0	2111	7	19.08	0.37	0.995	1.000
Chr1	9278	64	83.84	0.76	0.959	1.000
**Chr2**	**7080**	**124**	**63.98**	**1.95**	**<0.001**	**<0.001**
Chr3	7041	62	63.62	0.97	0.606	1.000
Chr4	5563	27	50.27	0.54	0.997	1.000
Chr5	5217	40	47.14	0.85	0.805	1.000
Chr6	4383	55	39.61	1.38	0.074	0.593
Chr7	4590	30	41.48	0.73	0.924	1.000
CC5 seed family						
Chr0	2111	12	14.08	0.86	0.722	1.000
Chr1	9278	37	61.90	0.60	0.996	1.000
**Chr2**	**7080**	**83**	**47.24**	**1.77**	**<0.001**	**0.008**
**Chr3**	**7041**	**84**	**46.98**	**1.80**	**<0.001**	**0.006**
Chr4	5563	14	37.12	0.38	1.000	1.000
Chr5	5217	34	34.81	0.97	0.595	1.000
Chr6	4383	18	29.24	0.62	0.961	1.000
Chr7	4590	20	30.63	0.64	0.954	1.000

Table S9. Summary of gene number for NTSR-related gene families. The percentage was presented in the parenthesis.

Species	P450	GST	ABC	UGT	AKR	Total NTSR	Total genes
A.thaliana	245 (0.89%)	55 (0.20%)	130 (0.47%)	107 (0.39%)	22 (0.08)	559 (2.04)	27420
O.sativa	356 (1.00%)	79 (0.22%)	133 (0.37%)	180 (0.50%)	29 (0.08%)	777 (2.18%)	35662
A.myosuroides	506 (1.17%)	93 (0.22%)	146 (0.34%)	278 (0.64)	46 (0.11%)	1069 (2.48%)	43152

Table S10. Summary of differentially expressed NTSR-related gene number in each blackgrass chromosome in CC2 and CC5 families.

Count of NTSR-related genes in each blackgrass chromosome						
**Chr**	P450	GST	ABC	UGT	AKR	Total
**1**	131	11	20	78	4	244
**2**	80	34	41	55	4	214
**3**	115	10	16	45	16	202
**4**	34	2	23	18	2	79
**5**	51	21	10	37	10	129
**6**	46	12	16	24	1	99
**7**	49	3	20	21	8	101
Count of differentially expressed NTSR-related gene in CC2						
**Chr**	P450	GST	ABC	UGT	AKR	Total
**1**	5	1	2	3	0	11
**2**	6	1	0	6	2	15
**3**	0	5	0	3	2	10
**4**	1	0	0	0	0	1
**5**	2	1	0	0	0	3
**6**	3	1	1	2	0	7
**7**	1	0	1	1	0	3
Count of differentially expressed NTSR-related gene in CC5						
**Chr**	P450	GST	ABC	UGT	AKR	Total
**1**	4	0	1	0	0	5
**2**	3	1	0	3	1	8
**3**	1	4	0	1	0	6
**4**	1	0	0	0	0	1
**5**	1	0	0	1	0	2
**6**	0	1	1	2	0	4
**7**	0	0	1	1	0	2

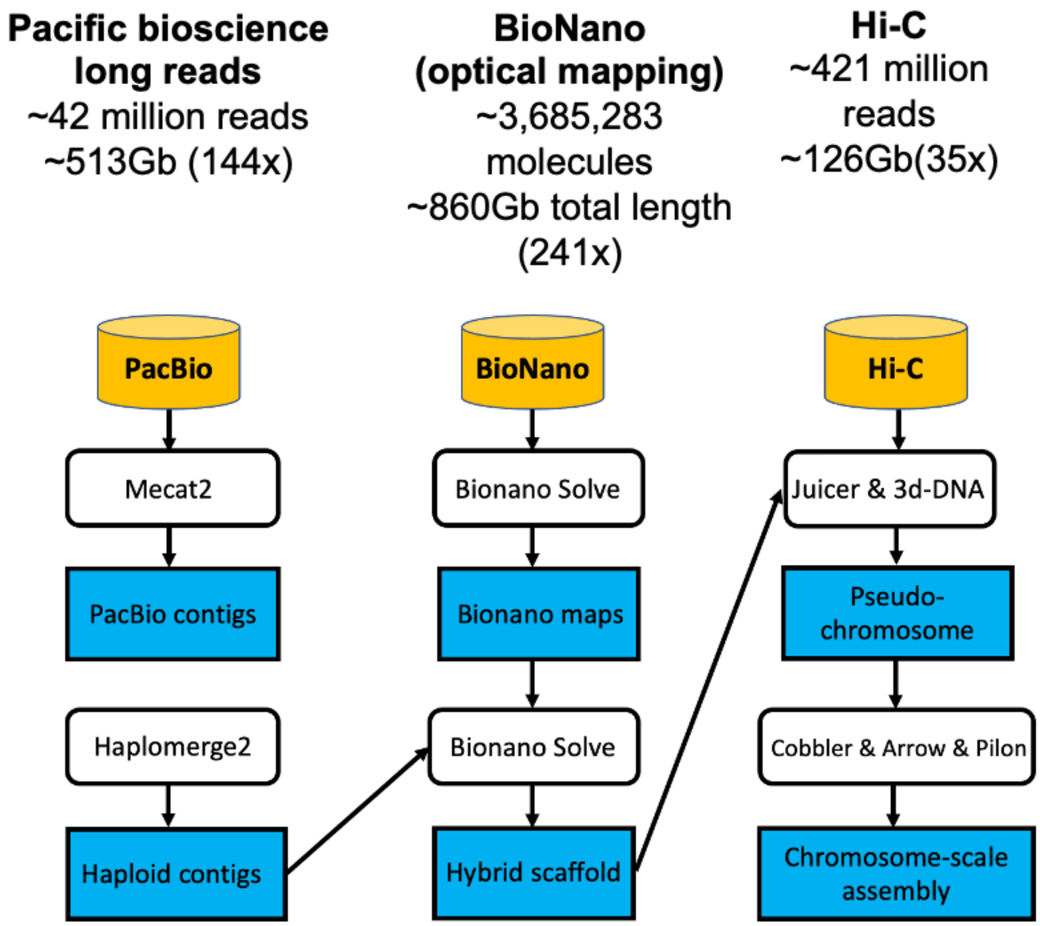

Fig. S1. Pipeline of genome assembly for the blackgrass.

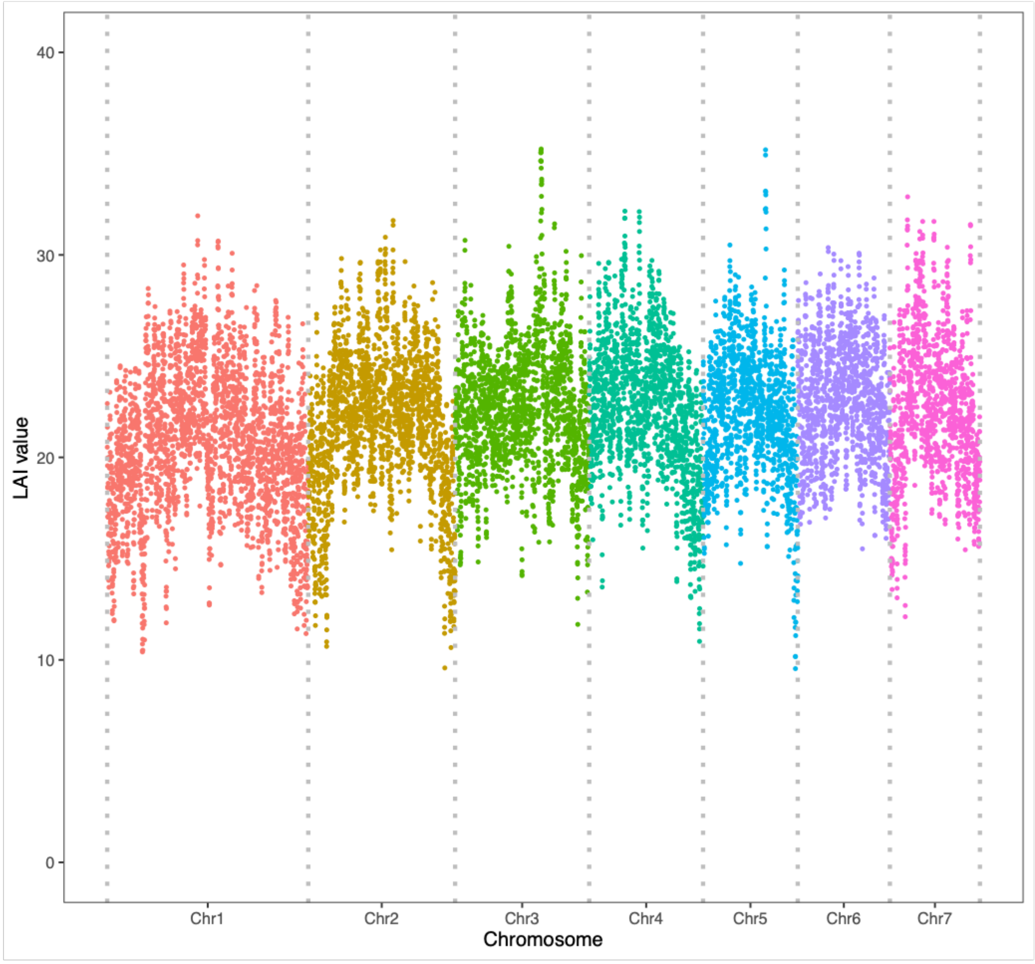

Fig. S2. Long terminal repeat assembly index (LAI) values across seven assembled blackgrass chromosomes.

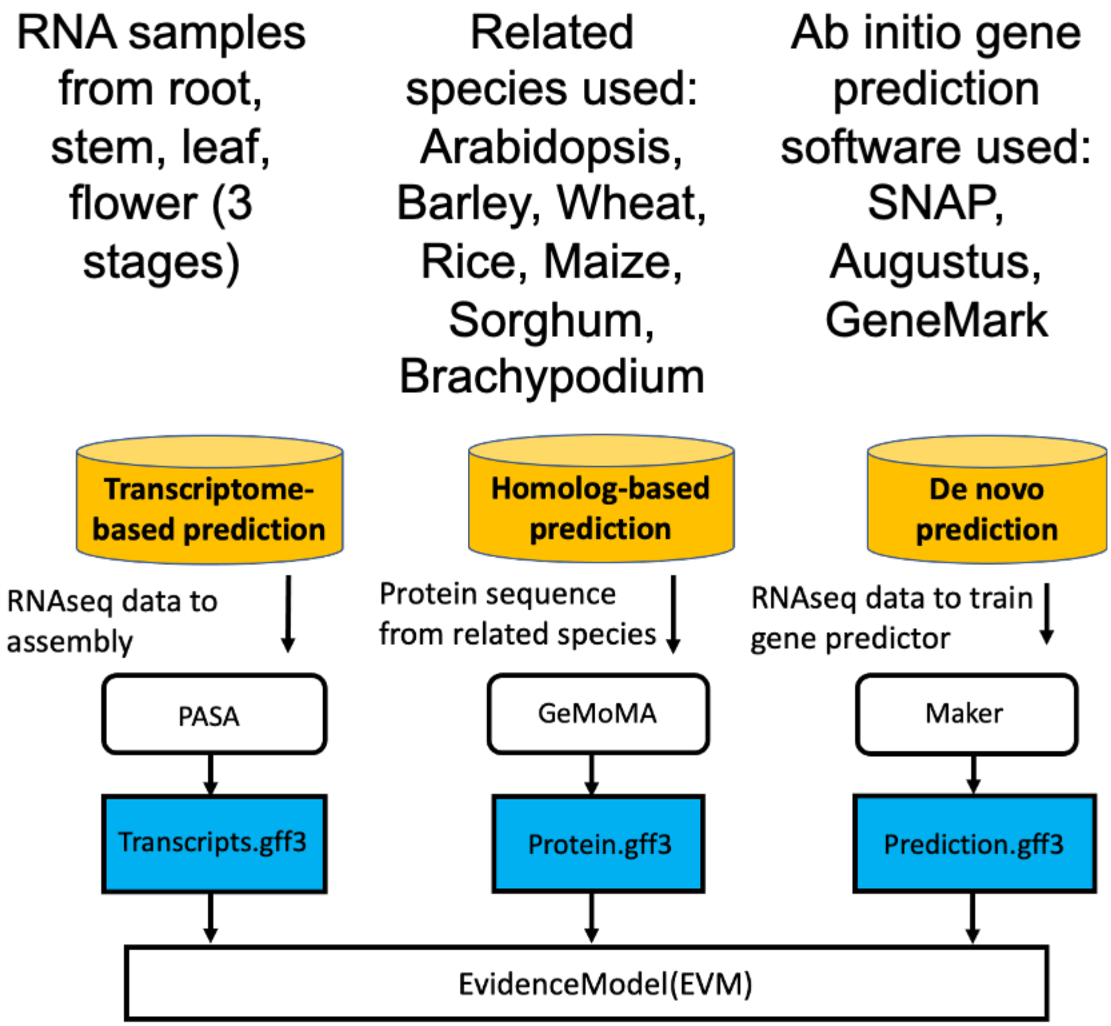

Fig. S3. Pipeline of gene annotation for the blackgrass.

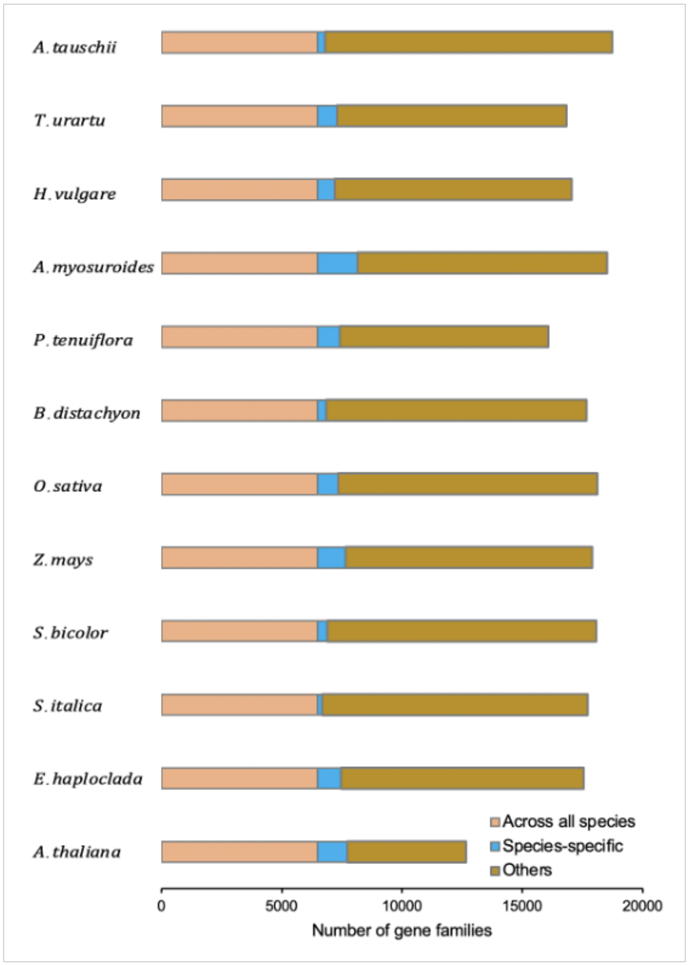

Fig. S4. The distribution of different types of gene family, including the ones presented across all the species, species-specific and all other types.

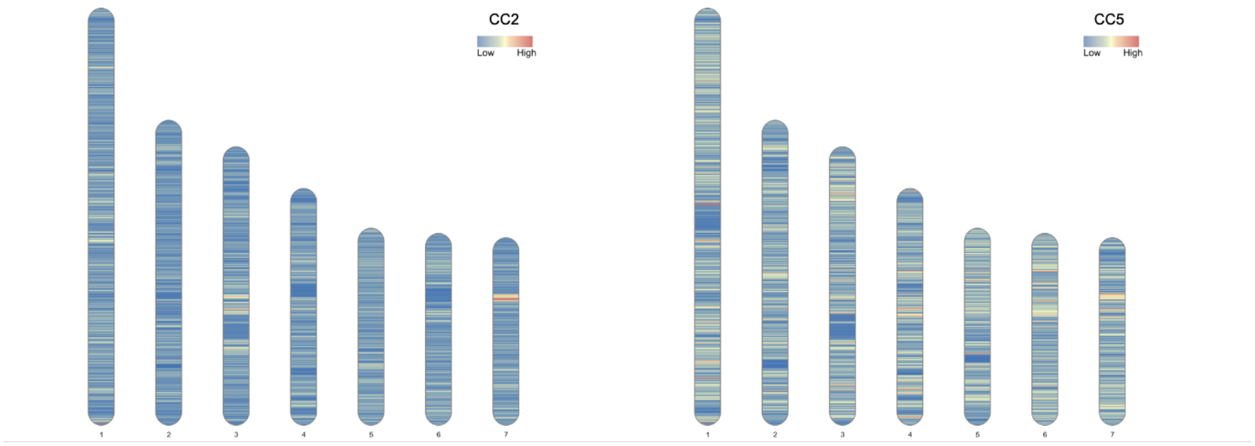

Fig. S5. SNP marker density for CC2 and CC5 population, respectively.

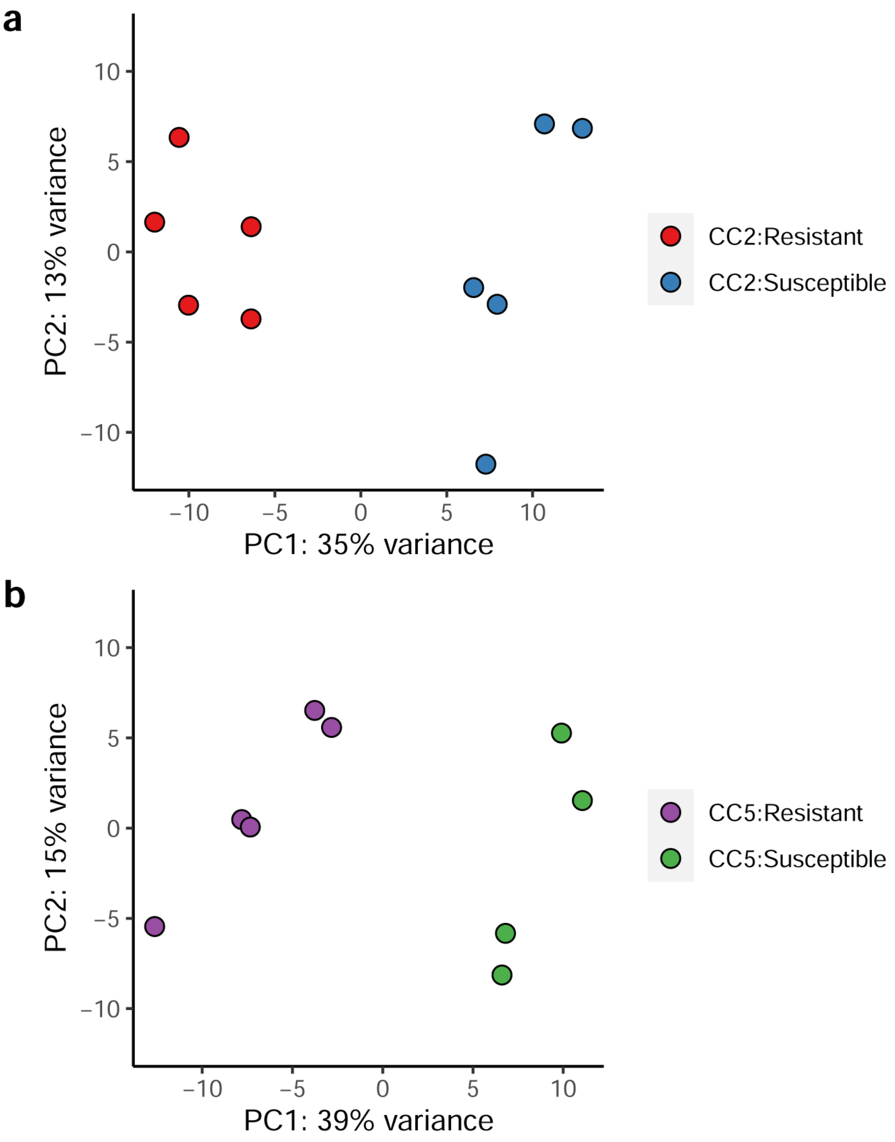

Fig. S6. Principal components analysis of all gene expression data for (a) the CC2 family and (b) the CC5 family alone.

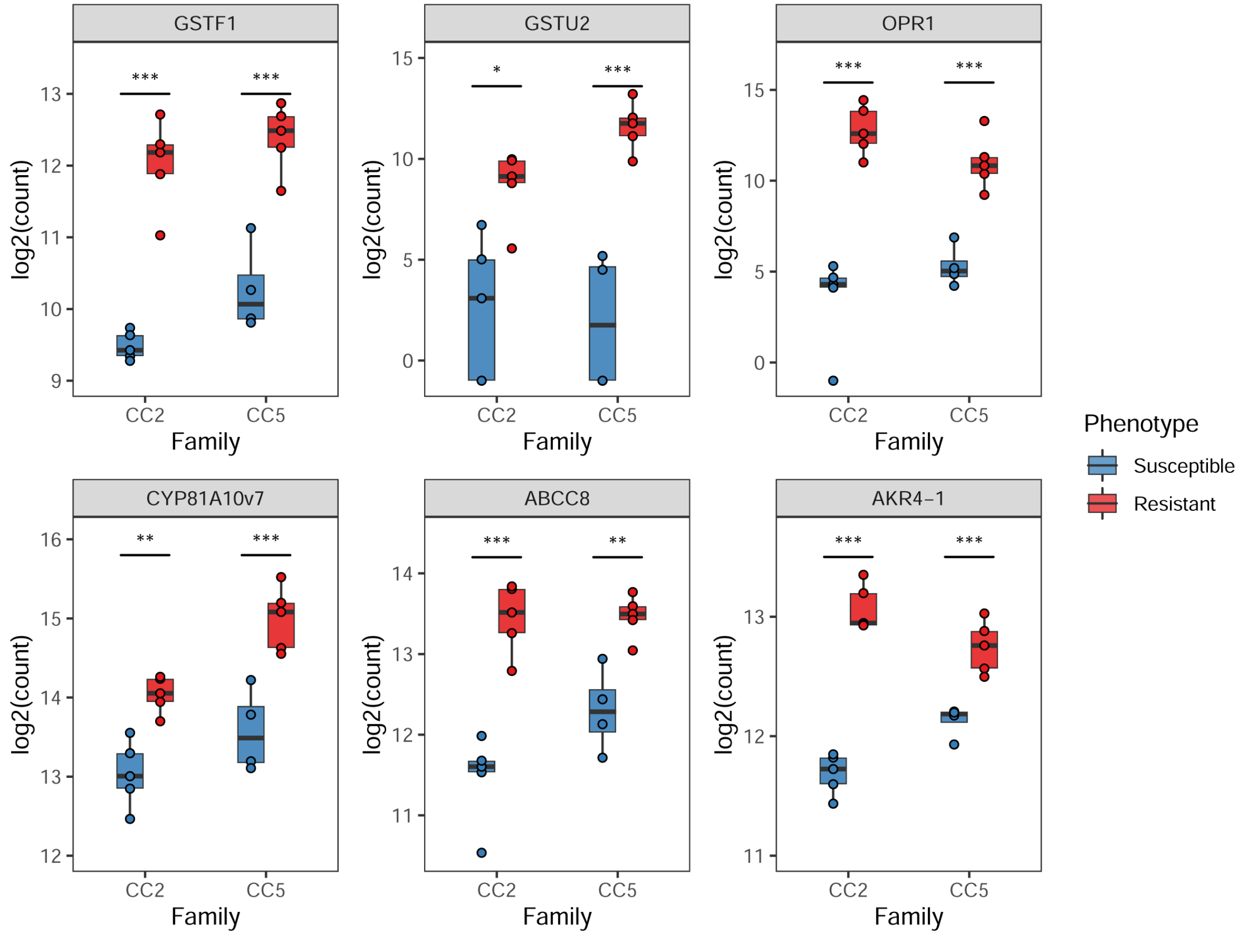

Fig. S7. Differential expression of previously reported NTSR candidate genes.

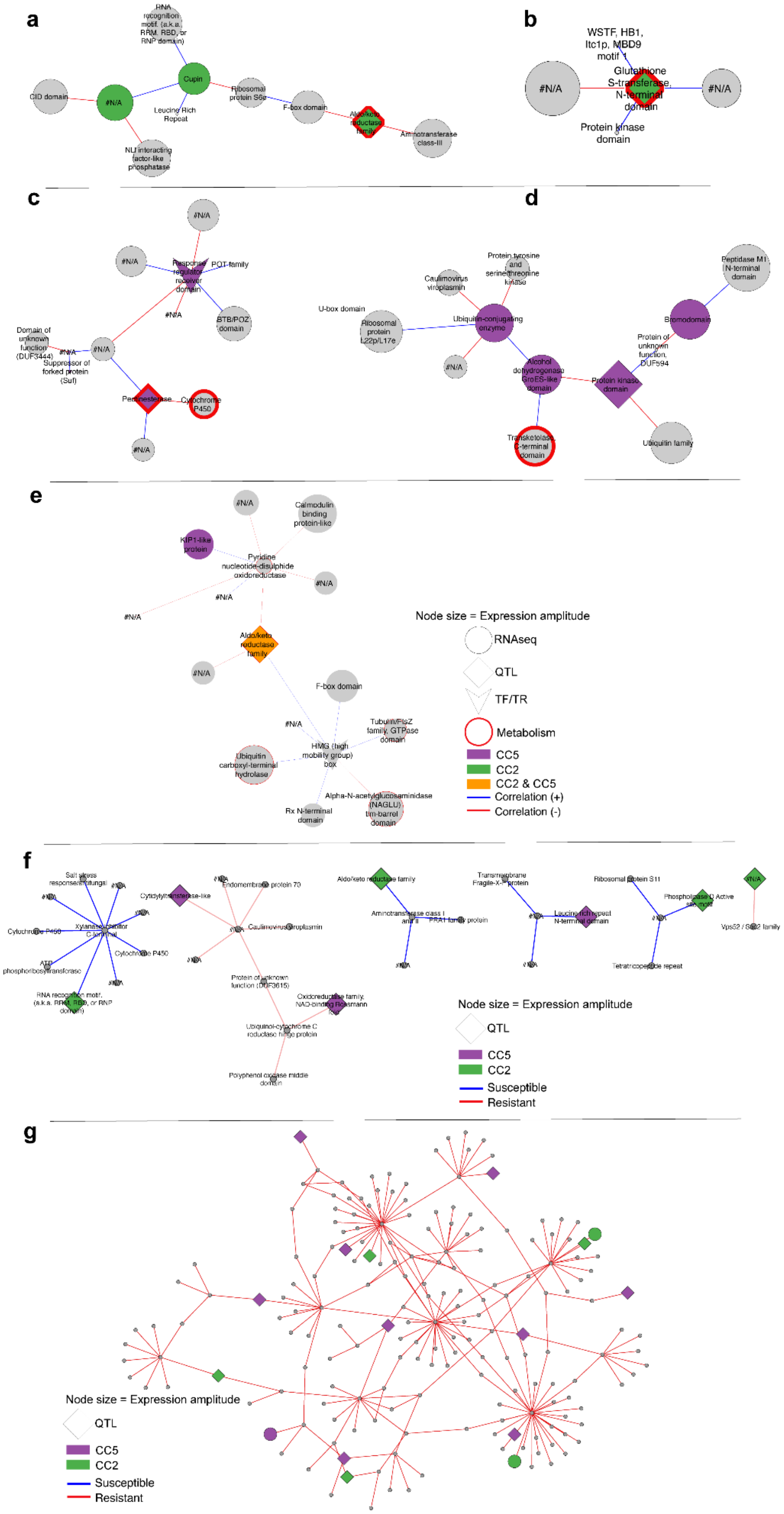

a
RNA recognition motif. (a.k.a. RRM, RBD, or RNP domain)
Cupin
CID domain
#N/A
Ribosomal protein S6e
Leucine Rich Repeat
F-box domain
Aldo/keto reductase family
Aminotransferase class-III
NLI interacting factor-like phosphatase
b
WSTF, HB1, Itc1p, MBD9 motif 1
Glutathione S-transferase, N-terminal domain
#N/A
Protein kinase domain
c
#N/A
Response regulator receiver domain
POT family
BTB/POZ domain
Domain of unknown function (DUF3444)
Suppressor of forked protein (Suf)
Pectinesterase
Cytochrome P450
d
Protein tyrosine and serine/threonine kinase
Caulimovirus viroplasmin
U-box domain
Ribosomal protein L22p/L17e
Ubiquitin-conjugating enzyme
Peptidase M1 N-terminal domain
Bromodomain
Protein of unknown function, DUF594
Alcohol dehydrogenase GroES-like domain
Protein kinase domain
Transketolase, C-terminal domain
Ubiquilin family
e
Calmodulin binding protein-like
KIP1-like protein
Pyridine nucleotide-disulphide oxidoreductase
Aldo/keto reductase family
F-box domain
Tubulin/FtsZ family, GTPase domain
HMG (high mobility group) box
Ubiquitin carboxyl-terminal hydrolase
Alpha-N-acetylglucosaminidase (NAGLU) tim-barrel domain
Rx N-terminal domain
Node size = Expression amplitude
RNAseq
QTL
TF/TR
Metabolism
CC5
CC2
CC2 & CC5
Correlation (+)
Correlation (-)
f
Cytidylyltransferase-like
Endomembrane protein 70
Caulimovirus viroplasmin
Cytochrome P450
Protein of unknown function (DUF3615)
Oxidoreductase family, NAD-binding Rossmann fold
Ubiquinol-cytochrome C reductase hinge protein
Polyphenol oxidase middle domain
Aldo/keto reductase family
Aminotransferase class I and II
PRA1 family protein
Transmembrane Fragile-X-F protein
Leucine rich repeat N-terminal domain
Ribosomal protein S11
Phospholipase D Active site motif
Tetratricopeptide repeat
Vps52 / Sac2 family
Node size = Expression amplitude
QTL
CC5
CC2
Susceptible
Resistant
g
Node size = Expression amplitude
QTL
CC5
CC2
Susceptible
Resistant

Fig. S8. Parallel and overlapping sub-graphs in CC2 and CC5. (a-b) Hub genes unique to CC2 subset from the traditional network discovered in the bulk-segregant analysis. (c-d) Hub genes unique to CC5 subset from the CC5 traditional network. (e) A hub gene that was discovered in both the CC2 bulk segregant analysis and is differentially expressed in resistant individuals in both families. (f) Representative condition-specific sub-graphs that display both independent and common gene-interaction pairs between CC2 and CC5 families. (g) A representative sub-graph from the condition specific network analysis that shows a large subgraph of genes identified in the bulk segregant analysis.

Notes S1. The development of research materials and genome sequencing and assembling.

1. The selection of sensitive individual plant for genome sequencing

To identify an herbicide sensitive blackgrass individual for genome sequencing, 16 plants from the 'Rothamsted' population were grown from seed within a glasshouse until they reached approximately six vegetative tillers in size. Plants were physically split into five tillers (vegetative clones), and aboveground biomass trimmed to a uniform ~5cm length. Each tiller was re-potted and allowed to recover for one week in the glasshouse. A separate tiller from each plant was sprayed with the herbicidal actives; Fenoxaprop-p-ethyl (69 g ai ha^{-1}), Cycloxydim (150 g ai ha^{-1}), Sulfometuron (133 g ai ha^{-1}), and a commercial formulation of the two actives mesosulfuron-methyl and iodosulfuron-methyl (12 and 2.4 g ai ha^{-1}), while one further tiller from each plant remained unsprayed. Four weeks after spraying, all sprayed tillers were visually inspected to confirm sensitivity to the herbicide. Since all plants were confirmed as equally herbicide sensitive, a single unsprayed tiller was chosen at random from the screened plants to use as the source of tissue for genome sequencing.

The chosen plant was cultivated under glasshouse conditions until it had formed a large vegetative mass. At this point the plant was physically split once more into 24 tillers, which were again allowed to recover within the glasshouse until they had re-established as large vegetative plants. To ensure that only young, healthy tissue was harvested for sequencing, these well-established vegetative clones were cut back a final time to approximately 5cm of aboveground growth. After three weeks, the healthy aboveground re-growth from these plants was harvested and immediately flash frozen in liquid Nitrogen. By sampling in this manner, a large quantity (>50g) of freshly expanded, healthy leaf tissue was harvested from a single blackgrass plant to sequence as the basis of the blackgrass genome. All tissue was stored at -80°C before homogenization in bulk using liquid Nitrogen to keep tissue frozen.

To aid genome annotation, further individuals of the Rothamsted population were maintained within the glasshouse to maturity, with tissue collected across a range of phenological stages.

Healthy leaf tissue was harvested, along with ‘stem’ material from between the top two nodes of the main tiller. Flower heads were collected at three developmental stages; developing, un-emerged inflorescences, mature emerged flower heads pre-anthesis, and mature flower heads with anthers visibly extended and producing pollen. Blackgrass root tissue was also sampled from hydroponically grown plants. Briefly, pre-germinated seeds of the Rothamsted population were transferred to foam/mesh inserts and placed within 8 x 8 x 10cm magenta boxes (n=5 boxes, with six seeds per box), filled with 100ml of hydroponic media (Long-Ashton solution). Boxes were kept within an incubator (Sanyo MLR-350) at 17/11°C temperatures over a 14/10 hour light/dark cycle, and hydroponic solution was changed weekly. After four weeks, all root material was harvested and immediately flash frozen in liquid nitrogen and kept at -80°C before use.

2. Genome Sequencing

Pacific Biosciences (PacBio) sequencing: high molecular weight (HMW) DNA was extracted from leaf tissues of a single plant (Rothamsted) that had been dark adapted for five days, used to construct PacBio SMRTbell libraries using SMRTbell Express Template Prep Kit 2.0, following the manufacturers’ protocols. SMRTbell libraries were sequenced on a PacBio Sequel II system and a total of six SMRT cells and 513 Gb (144 X coverage) data composed of ~42 million reads were generated.

BioNano optical maps: HMW DNA was isolated from the same leaf tissue according to the BioNano Prep Plant Tissue DNA isolation protocol, and then fluorescently labelled using single-sequence-specific DLE1 endonucleases based on BioNano’s Direct Label and Stain (DLS) technology. The labelled DNA was loaded on the BioNano Genomics Saphyr system to scan by the sequencing provider. A total of 3,685,283 BioNano molecules were obtained with a total length of 860 Gb (241 X coverage).

Chromosome conformation capture sequencing by Hi-C: chromatin conformation capture data was generated using a Phase Genomics (Seattle, WA) Proximo Hi-C 2.0 Kit. Following the manufacturer's instructions for the kit, intact cells from two samples were crosslinked using a

formaldehyde solution, digested using the Sau3AI restriction enzyme, and proximity ligated with biotinylated nucleotides to create chimeric molecules composed of fragments from different regions of the genome that were physically proximal in vivo, but not necessarily proximal in the genome. Continuing with the manufacturer's protocol, molecules were pulled down with streptavidin beads and processed into an Illumina-compatible sequencing library. Sequencing was performed on an Illumina HiSeq 4000 system, yielding 126 Gb (35 X coverage) data.

Illumina short reads for polishing: DNA was extracted with the DNeasy Plant Mini Kit (QIAGEN) to prepare PCR-free paired-end libraries using the Illumina Genomic DNA Sample Preparation kit following the manufacturer's instructions (Illumina). All paired-end libraries were sequenced on an Illumina NovaSeq 6000 system, generating 291 Gb (81 X coverage) of 150-nucleotide paired-end reads.

3. Genome assembling

We adopted a hierarchical sequencing approach to generate a high-quality reference genome for blackgrass from a single individual using PacBio long reads, BioNano optical maps, chromatin conformation capture sequencing (Hi-C) and paired Illumina short reads (Supplementary Figure 1). First, we *de novo* assembled PacBio long reads into 12,107 contigs resulting in a total size of 4,906 Mb. After polishing, haplotype identification and deduplication, we obtained a primary contig set (representing homozygous regions of genome) that includes 7,866 contigs with an N_{50} of 1.2 Mb. The total primary contig length is 3,475 Mb, which is consistent with our genome survey estimations based on flow cytometry and k-mer analysis (3,312-3,423 Mb and 3,400-3,550 Mb, respectively). BioNano data were first filtered based on molecule length (> 150Kb) and then aligned to primary contigs to select mapped molecules for de novo assembly, which produced 3,530 genome maps with an N_{50} of 1.8 Mb for a total map length of 4367 Mb. Second, the primary contigs were corrected for false joins and scaffolded with optical map alignments (BioNano) to produce 2,512 hybrid scaffolds with the longest scaffold at 17.7 Mb. This increased the contiguity as indicated by N_{50} value from 1.2 Mb to 2.3 Mb. Finally, hybrid scaffolds were ordered and oriented with Hi-C three-dimensional proximity data (Notes S1 Figs. 1-2). The final polished blackgrass genome assembly size was highly complete and contiguous at

3,572 Mb, including 3,400 (95.2%) Mb ordered as seven chromosomes with only 172 Mb of unanchored sequences.

4. Generating F_2 families

To explore the genetic basis of herbicide resistance in Blackgrass, two F_2 seed families were generated, with segregating non-target-site resistance (NTSR) to the Acetyl-CoA-Carboxylase inhibiting herbicide group (ACCase). To do this, plants from the herbicide susceptible Rothamsted population were grown in a glasshouse, along with individuals from two field-collected Blackgrass seed populations previously characterized as being strongly NTSR to the ACCase herbicides (Peldon, and Lola91). Plants were maintained for 2-3 months until well established. A single plant was chosen from each population and vegetatively cloned (tillering). The resistance phenotype was confirmed for these individuals by spraying one set of clones from each plant with a discriminatory dose of the herbicide Fenoxaprop-P-ethyl. The absence of ACCase target-site-resistance (TSR) in the selected plants was further confirmed by long-read sequencing of the ACCase gene.

To produce the F_1 generation, two clones of the herbicide sensitive individual from the Rothamsted population, and the resistant individual from each of the 'Peldon' and 'Lola91' populations, were vernalized for two months over winter in an unlit, unheated glasshouse. In the following spring, plants were paired such that each of the herbicide sensitive clones was paired with one of the two herbicide resistant individuals. Paired plants were moved to separate corners of a large glasshouse chamber, and before flowering, each pair was enclosed in a pollination bag. During flowering, bags were very gently shaken to assist pollen movement, facilitating cross-pollination of the two individuals enclosed. Once seed had matured, the pollination bags were removed, and seeds collected by gently shaking seed-heads into a paper envelope. As blackgrass is an obligate outcrossing species, seeds derived were the product of pairwise crossing between the enclosed pair of plants.

Collected seeds represent the F_1 generation of these two pairwise crosses. Seeds were air-dried at room temperature and cleaned using an air-column seed cleaner. Cleaned seeds were incubated

at 30°C for three weeks to break dormancy. To generate the segregating F_2 families, 20 plants were grown from each of the F_1 lines. Plants were kept outside over Autumn – Spring allowing vernalization and vegetative establishment. Before flowering, the two sets of plants were moved into separate small pollination glasshouses. Plants were allowed to flower and openly cross-pollinate within each glasshouse, before collecting seeds in bulk from all individuals within each line. As before, these seeds were dried, cleaned and incubated at 30°C to break dormancy, and represent the F_2 segregating families used in the subsequent QTL-seq and RNA-seq analyses. Seed families were given the experimental codes 'CC2' (F_2 derived from Rothamsted x Peldon), and 'CC5' (F_2 derived from Rothamsted x Lola91). For an overview of F_2 line creation and subsequent bulk-segregant analysis, see Notes S1 Fig. 3.

5. Material for bulked segregant analysis

For each of the families (CC2 and CC5), 600 plants were grown under glasshouse conditions until well established, and split into separate vegetative tillers. For 300 plants in each family, two tillers were sprayed with a 'low' dose of the herbicide Fenoxaprop-p-ethyl. Plants which were consistently killed at this low dose represent herbicide susceptible individuals. Tillers for the remaining 300 plants of each family were sprayed with a 'high' doses of the herbicide. Consistent survivors of this high dose represent individuals with herbicide resistance. Herbicide doses were tailored to the two F_2 families based upon the resistance status of their original field-collected parental 'R' populations. For the CC2 family, doses of 69 g ai ha^{-1} and 1104 g ai ha^{-1} (1 and 16x the UK field rate) were found to optimally discriminate between the least- and most-resistant individuals, while for the CC5 family, doses of 138 g ai ha^{-1} and 1656 g ai ha^{-1} (2 and 24× field rate) were used. A single tiller from a further 24 randomly chosen plants in each line were sprayed at field rate with the herbicide cycloxydim, to provide an additional confirmation of the absence of TSR within these lines.

Plants were visually inspected on several occasions over the subsequent three weeks, and ranked to identify the 30-40 plants most affected by the 'low' doses (i.e. killed the quickest or at the smallest size), and least affected by the 'high' herbicide doses (i.e. those with vigorous growth matching the unsprayed tiller). At each dose, both tillers from the 30-40 chosen individuals were

harvested, alongside both tillers from a further 100 randomly chosen plants. Aboveground ‘fresh’ biomass was recorded for each tiller, with dry biomass recorded following oven drying at 80°C for 48 hours. Biomass measurements were used in combination with the visual scoring information to select the 25 plants either most susceptible (‘S’), or most resistant (‘R’) to the herbicide within each family. Leaf material from the un-sprayed clones of these chosen ‘R’ and ‘S’ plants was harvested, flash frozen in liquid Nitrogen, and used throughout the RNA-seq and QTL-seq bulked segregant analyses (Notes S1 Fig. 3).

6. Confirming resistance status of the F_2 families

A glasshouse phenotyping assay was used to confirm the resistance status of the F_2 seed lines in comparison to their original parental populations. Seeds of both the ‘CC2’ and ‘CC5’ F_2 families, as well as the original ‘Rothamsted’, ‘Peldon’, and ‘Lola91’ populations, were germinated in Petri dishes containing three Whatman No. 1 filter papers soaked in 5 ml of 0.02 M KNO3. Petri dishes were incubated for seven days in a Sanyo MLR-350 growth cabinet, with a 17/11°C temperature cycle and a 14/10-hour light/dark cycle. Germinated seeds were transplanted 5 – 10mm below the soil surface in 8 cm plastic plant pots (six seeds per pot), filled with a Kettering loam soil mixed with 2 kg m-2 osmocote fertilizer. Plants were maintained in a glasshouse at approximately 16/10°C until seedlings had reached the three-leaf stage, before spraying with the ACCase herbicide Fenoxaprop-P-ethyl (using the commercial formulation ‘Polecat’). Doses used were: 0, 2.16, 4.31, 8.63, 17.25, 34.5, 69, 138, 276, 552, and 1104 g ai ha^{-1}, representing 0, 0.03, 0.06, 0.125, 0.25, 0.5, 1, 2, 4, 8, and 16x the UK field rate for this active. Herbicide was applied using a fixed track sprayer, with a spray nozzle (flat fan 110015VK; Teejet, Wisbech, UK) mounted 50 cm above the pots, and boom speed set at 0.33 m s^{-1}, applying herbicide at a rate of 199 l ha^{-1}. Three pots of each population (n=3) were sprayed per dose, and pots were placed back in the glasshouse immediately following spraying. Three weeks after herbicide application, all plants were assessed for mortality, and above-ground leaf and shoot biomass was harvested, dried at 80˚C for 48 hours, and weighed. Dose-response relationships were compared for each F_2 line relative to their parent populations. In both cases, the phenotypic level of herbicide resistance in the two F_2 families was intermediate, between that of the resistant and susceptible

parental families, as expected for inheritance of this potentially quantitative trait (Notes S1 Fig. 4, a-b).

7. Confirming the 'R' and 'S' phenotypes in the F_3 generation

After tissue sampling, the 25 confirmed 'R' and 'S' plants from the CC2 and CC5 F_2 families were re-potted into larger, 6-inch pots, and grown to maturity. At the onset of flowering, plants were moved into four small pollination glasshouses, with all 25 plants from the same phenotypic group (CC2 'R', CC2 'S', CC5 'R', CC5 'S') kept together. Flowering heads were agitated once per week to help pollen movement and facilitate bulk cross pollination within each glasshouse. Seeds were collected at maturity and air dried at room temperature before cleaning with an air column seed cleaner to remove empty husks and debris. Cleaned seeds were incubated at 30°C in a Sanyo MLR-350 growth cabinet for three weeks to break dormancy.

These seeds represent F_3 lines from the CC2 and CC5 families. If the 'R' and 'S' plants identified from the F_2 truly represent individuals with genetic segregation for the herbicide resistance trait, we would expect that a difference in 'R' and 'S' phenotype would be maintained into this F_3 generation. To confirm this, seeds from these 'R' and 'S' F_3 lines, along with the F_2 CC2 and CC5 lines that they were derived from, were tested under glasshouse conditions for their Fenoxaprop-P-ethyl dose-response relationship. Seeds were germinated in Petri dishes containing three Whatman No. 1 filter papers soaked in 5 ml of 0.02 M KNO3. Petri dishes were incubated for seven days in a Sanyo MLR-350 growth cabinet, with a 17/11°C temperature cycle and a 14/10 hour light/dark cycle. Six germinated seeds were transplanted 5 – 10mm below the soil surface into an 8 cm plastic plant pot containing a Kettering loam soil mixed with 2 kg m-2 osmocote fertilizer.

Pots were kept in a glasshouse for approximately three weeks until plants had reached the three-leaf stage, and were then sprayed with Fenoxaprop-P-ethyl using the same commercial formulation as previously (Foxtrot). Doses used were 0, 1.08, 2.16, 4.31, 8.63, 17.25, 34.5, 69, 138, 276, 552, 1104, and 2208 g ai ha^{-1}, and for each line, three replicate pots were sprayed per dose. The same fixed track-spraying unit and Teejet 110015VK nozzle were used, as previously

described. Three weeks after spraying, plants were visually assessed for survival and dose-response relationships compared. Comparison of herbicide survival confirms that the expected 'R' and 'S' phenotype is maintained within these 'R' and 'S' F_3 lines (Notes S1 Fig. 4, c-d).

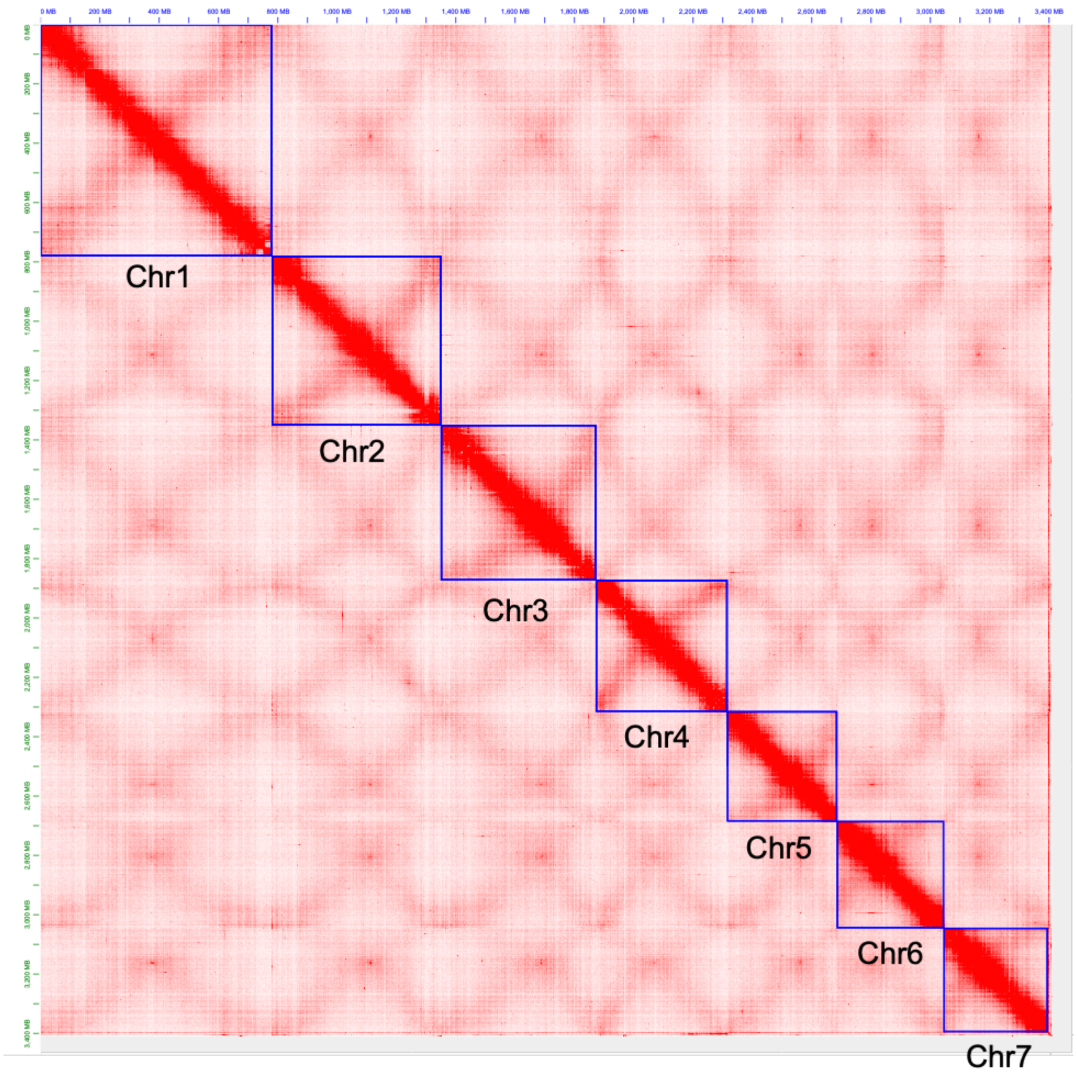

Notes S1 Fig. 1. Snapshot of Juicebox Assembly Tools output after manual correction.

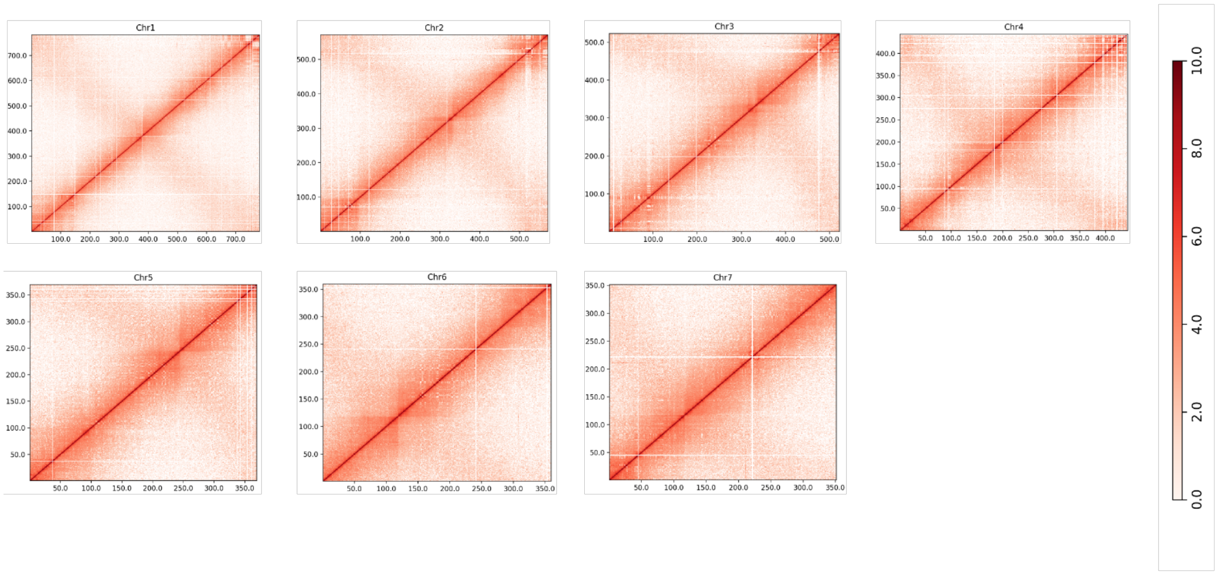

Notes S1 Fig. 2. Genome-wide analysis of chromatin interactions at 1-Mb resolution in the assembled blackgrass genome.

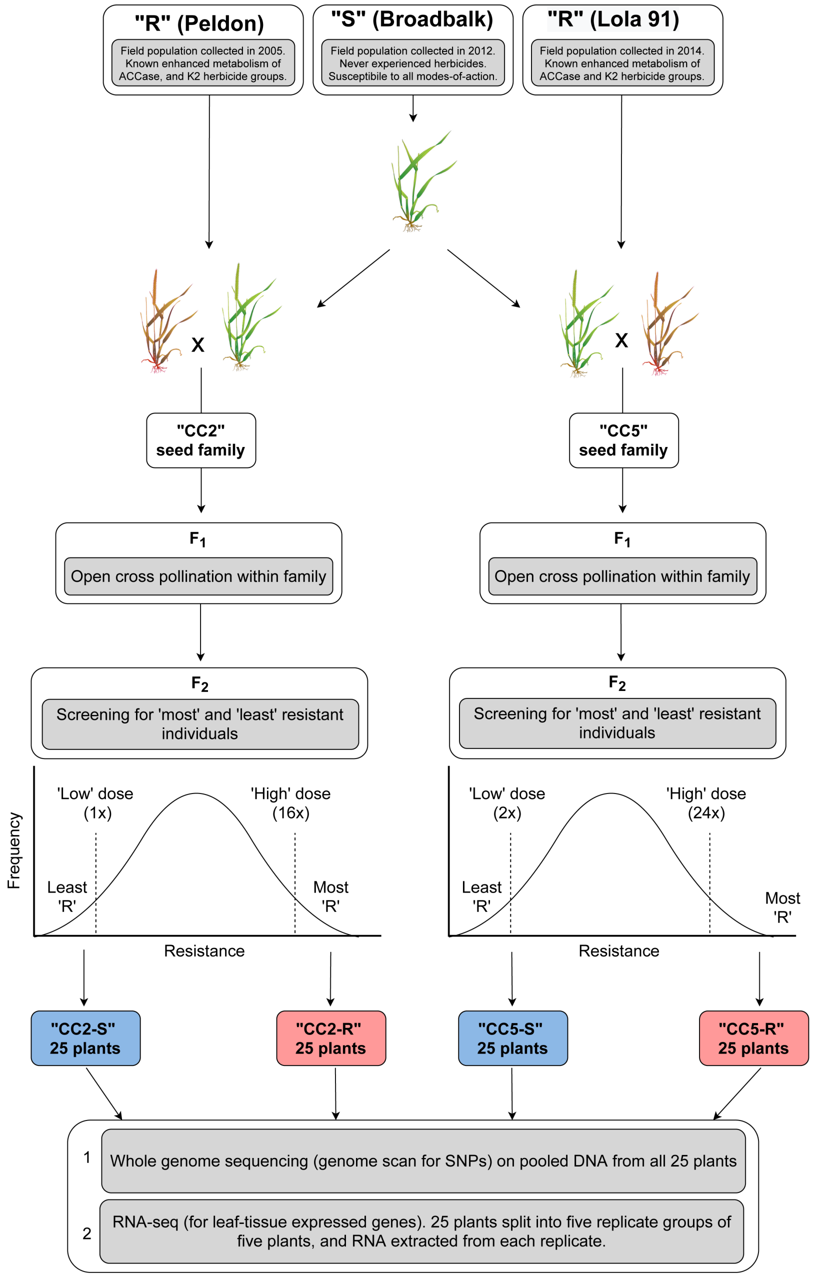

Notes S1 Fig. 3. Overview of the steps involved in creating the experimental seed families. Initially a single 'S' plant of the Broadbalk population was vegetatively cloned and cross pollinated with an individual from one of the two 'R' populations. Herbicide screening in the F_2 generation was performed using the ACCase inhibitor Fenoxaprop-P-ethyl. Tissue was collected from 25 plants identified as either 'R' or 'S' in each family, and used for whole genome sequencing and analysis of gene expression.

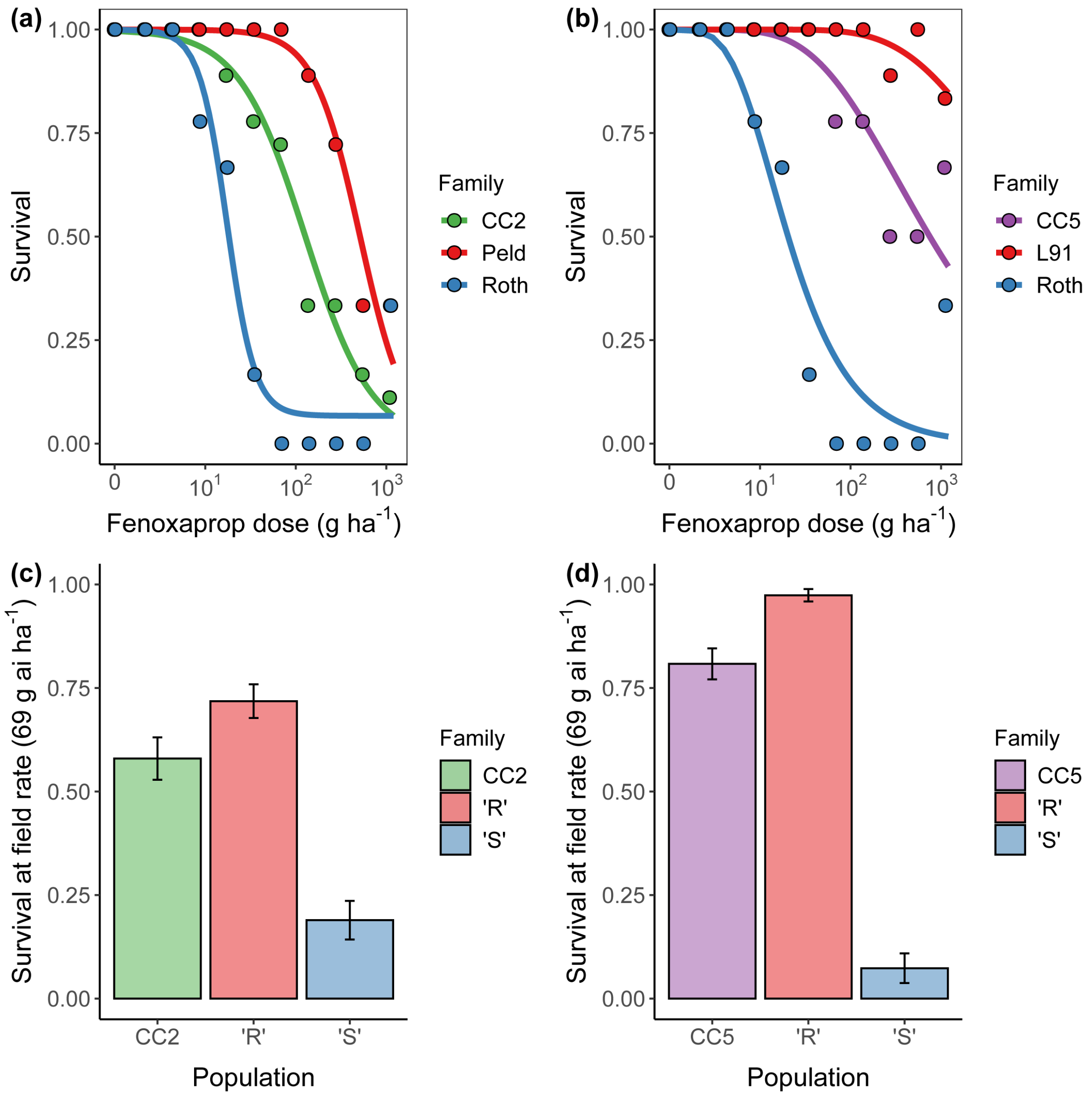

Notes S1 Fig. 4. Confirmation of resistance phenotype in the experimental seed families. **a** and **b** show the dose-response relationship of the two F_2 seed families (CC2 and CC5), relative to their respective parental populations. **c** and **d** show herbicide resistance of progeny from the 'R' and 'S' bulks, relative to the seed family from which they were derived. In all cases resistance was determined using a commercial formulation of the Acetyl CoA carboxylase inhibiting herbicide fenoxyprop-P-ethyl.
